# Identification of Proteins Associated with Stably Integrated Maize *b1* Tandem Repeat Transgene Chromatin

**DOI:** 10.3390/plants14121863

**Published:** 2025-06-17

**Authors:** Jason S. Lynn, Kathryn M. Koirtyohann, Yacob B. Gebreab, Jaliyah Edwards, Karen M. McGinnis

**Affiliations:** Department of Biological Science, Florida State University, Tallahassee, FL 32304, USA; jlynn@cshl.edu (J.S.L.); kkoirtyohann@fsu.edu (K.M.K.); ygebreab@bio.fsu.edu (Y.B.G.); jedwards4@fsu.edu (J.E.)

**Keywords:** ChIP-mass spec, maize, proteomics, paramutation, transcriptional gene silencing, Pol II

## Abstract

The control of gene expression by *cis*-regulatory DNA sequences is a conserved genomic feature. The maize *booster1* gene (*b1*) is a naturally occurring locus that serves as a mechanistic model for the control of gene expression from a distal *cis* element and a form of allelic interactions called paramutation. Two epi-alleles of *b1* produce distinct pigmentation phenotypes correlated with transcriptional enhancement and the silencing of *b1*. These transcriptional dynamics depend on a hepta-tandem repeat sequence located 100 kb upstream of the *b1* locus. In the heterozygous condition, the *B*′ epi-allele paramutates *B-I*, heritably converting the *B-I* epi-allele to the epigenetic state and expression level of *B*′, producing lightly pigmented plants. To identify *b1TR*-associated proteins, we used a targeted chromatin immunoprecipitation approach with a stably integrated transgenic *b1TR* locus. Applying a conservative filtering strategy, we detected several expected factors, including RNA Polymerase II, as well as the novel putative DNA-binding proteins ZAG4 and DDT4. ZAG4 and DDT4 activated GAL expression using *b1TR* as bait in yeast one-hybrid, supporting their potential interaction with this sequence. The identification of proteins uniquely associated with the *UAS::b1TR* chromatin provides insight into potential *b1* regulatory factors and offers a foundation for future studies to investigate their roles in gene regulation.

## 1. Introduction

Transcription is spatiotemporally controlled, requiring the contribution of DNA regulatory sequences and chromatin-interacting proteins. Historically, *cis*-regulatory elements have been studied via genetic approaches, such as sequence deletions to test for necessity, and by the insertion of those sequences at heterologous genomic or reporter gene locations to test for sufficiency (reviewed in [1]). The investigation of DNA–protein interactions has been performed using in vitro or heterologous assays, such as the yeast one-hybrid assay or DNA-affinity purification [2,3], which can identify proteins that bind to *cis*-elements and determine the minimal sequences required for interaction. However, none of these methods maintain the in vivo context of chromatin and are therefore not necessarily representative of the relevant biochemical context of the regulatory sequences.

Chromatin-immunoprecipitation DNA-sequencing (ChIP-seq) and related methods are widely used to map the binding sites of a wide range of chromatin-interacting proteins, such as transcription factors and histone modifications. These approaches query the DNA associated with specific proteins and do not allow for the de novo identification of unknown proteins interacting with specific *cis* elements. To better understand the role of specific proteins at a particular *cis*-regulatory element, locus-specific chromatin capture-based proteomic methods are required. A few methods have been published seeking to identify proteins associated with well-characterized genetic elements in model organisms and cell lines [4,5,6,7]. More recent attempts to identify single-locus proteomes using modified CRISPR/Cas9 methods have allowed the characterization of protein factors at some chromosomal sequences [8,9,10,11,12]. Most of these approaches have been focused on characterizing proteins associated with large, highly repetitive centromere and telomere regions rather than low copy or unique loci.

The maize *b1 hepta tandem repeat* (*b1TR*) is a tissue-specific *cis*-regulatory element located on the short arm of chromosome 2 that can exist in distinct states associated with two genetically identical, but epigenetically distinct, alleles of *b1* referred to as *B-Intense* (*B-I*) and *B-Prime* (*B*′). In the *B*′ configuration, the hepta-repeat *b1TR* acts as a silencer, leading to low B1 expression and lightly-pigmented sheath and husk tissue; in the *B-I* configuration, the hepta-repeat *b1TR* is in a distinct epigenetic state and acts as an enhancer leading to high B1 expression and deeply-pigmented sheath and husk tissue [13,14,15,16]. This phenomenon is subject to genetic variation, with copy number variation of naturally occurring and transgenic versions of the *b1TR* sequence playing a key role in epigenetic regulation and inheritance. Alleles of *b1* with less than three repeats at *b1TR* do not display enhancer or silencer activity while five or more repeats at *b1TR* are required for transcriptional enhancement and the paramutation of *b1* (reviewed in [17]). The expression of the *b1* gene is correlated with changes in the epigenetic state of *b1TR* [16] and differential tissue-specific long-range intra-chromosomal contacts [15]. Although the establishment of paramutation is expected to occur during meiosis (Coe 1959 [18]), the transmission of this epigenetic state must be maintained throughout development because the *B*′ allele remains transcriptionally silent in the sheath and husk tissues of the mature plant [19]. Genetic analysis shows that proteins predicted to be components of the RNA-directed DNA-methylation pathway (RdDM) are differentially required for the establishment and maintenance stages of paramutation, meaning that different individual complexes and proteins might bind to and act on *b1TR* at different stages of paramutation [20,21,22,23,24,25,26,27,28].

The identification of *b1TR*-interacting proteins will therefore be important in understanding the mechanisms underlying these distinct regulatory processes. Transgenic versions of *b1TR* are sufficient for the silencing and paramutation of the endogenous *b1TR* enhancer from non-allelic positions [29], suggesting that *b1TR*-interacting regulatory proteins bind transgenic versions of this sequence. To identify proteins that interact with *b1TR*, we used a transgene-mediated chromatin-immunoprecipitation mass-spectrometry-based proteomics (ChIP-MS) approach that utilizes the yeast-derived GAL4 DNA binding protein’s specificity for its binding site and the Upstream Activation Sequence (GAL4-UAS) [30] to enrich for *b1TR*-sequence-associated chromatin-interacting proteins. We show that a transgenic copy of b1TR cloned adjacent to GAL4-UAS (*UAS::b1TR* transgene) is sufficient to induce silencing of *B-I* in stably transformed plants. Using this ChIP-MS approach, we detected peptides of components of the Mediator complex and the RNA Polymerase II transcriptional complex and generated a list of RNA- and DNA-binding proteins that uniquely associate with the *UAS::b1TR* chromatin. Two of these putative DNA binding proteins, DDT4 and ZAG4, were able to activate transcription of the GAL reporter locus using *b1TR* as bait in a yeast one-hybrid assay.

## 2. Methods

### 2.1. Maize Plasmid Construction

The *UAS::b1TR, UAS::empty*, and *FLAG-GAL4* constructs were generously gifted by Vicki Chandler, Jan Brzeski, and Kasia Brzeska. The *UAS::b1TR* construct was generated by fusing the *b1TR* sequence (7 × 853 bp tandem repeat) from the pBΔ construct [29] to an array of ten yeast minimal *GAL4-UPSTREAM ACTIVATION SEQUENCE* (UAS; 10 × 90 bp UAS) corresponding to the NCBI accession CP036475.1 (*Saccharomyces cerevisiae* strain ySR128; Chr13:599010-599024). The *UAS::empty* construct was generated by excising the 6684 bp hepta-*b1TR* sequence from the *UAS::b1TR* construct by using restriction digest and re-ligation. The *FLAG-GAL4* construct encodes an N-terminal-FLAG:GAL4 DNA binding domain corresponding to the NCBI accession CP033485.1 (*S. cerevisiae* strain y169; Chr16:81014-81471) and the Alcohol dehydrogenase 1 (*adh1*) intron. Expression is driven by the maize *ub1* promoter and is terminated by the OCS 3′ signal sequence. Additionally, all constructs contain glufosinate resistance under the control of the minimal Cauliflower mosaic virus 35S promoter (CaMV 35S) and the Tobacco mosaic virus Omega leader and are terminated by the nopaline synthase polyadenylation signal (NOS).

### 2.2. Transformation of Maize Stocks

The transformation of maize (*Zea mays*) plants was performed by the Iowa State University Plant Transformation Facility using biolistic bombardment of *Hi-II* embryos [31]. Glufosinate resistant calli were screened for transgene insertion by PCR. Plantlets were regenerated and backcrossed with *Hi-II* to generate plants with single transgene insertions. Transgenic *Hi-II* plants were crossed to *B-I/B-Peru* plants to generate segregating progeny for the transgene and *B-I*. We refer to the *B* allele of *Hi-II* as *B-neutral* (*b-N*) because it does not confer intense pigmentation to the sheath and husk tissues nor does it participate in paramutation.

### 2.3. Pigmentation Analysis of the UAS::b1TR and UAS::Empty Transgenes for Silencing of B-I

The silencing abilities of *UAS::b1TR* constructs were determined by pigmentation scoring of sheath and husk tissues in a blind, randomized field experiment. A total of 179 transgenic plants containing *UAS::b1TR* and 203 non-transgenic plants were segregating for *B-I*/*b-N* or *B-Peru/b-N*. 167 transgenic plants containing *UAS::empty*, and 158 non-transgenic plants were segregating for *B-I/b-N* or *B-Peru/b-N*. *B-I* and *B*′ plants were used as controls for herbicide scoring and pigmentation. In addition to *b1*, a functional copy of *Pl1* and the full complement of anthocyanin biosynthetic genes were required for complete pigmentation. All plants contained at least one *Pl1* allele, conferring strong pigmentation as *B-I/B-Peru* parents were homozygous for *Pl1*. Each individual plant was numbered and tagged, and the genotypic identity of the families was hidden from observers. At maturity (R1, silks exposed), the plants were scored for sheath and husk color by three independent blinded observers using a color scale of 1–5 with 5 being darkest. Plants with a color score <4 were considered light, while plants with a color score of ≥4 were considered pigmented. Finale^®^ herbicide (1.6% *v*/*v* in water; BASF) was applied to a leaf from every individual to differentiate transgenic and non-transgenic plants by scoring for the presence or absence of leaf tissue damage from herbicide application. Any observations that produced a standard deviation ≥1 for either sheath- or husk-color score across three independent observers were considered erroneous and were removed from subsequent analysis.

### 2.4. Sample Growth Conditions and Sample Collection

Maize plants were grown in a greenhouse with supplemental lighting to extend the day length. Finale^®^ herbicide (1.6% *v*/*v* in water; BASF) was applied to the leaves to screen for transgenic plants with a glufosinate resistance gene. Herbicide-resistant plants were genotyped using transgene-specific primers KM1684 (5′-GCGCTTTCTCATAGCTCAC-3′) and KM1687 (5′-AGTCGTGTCTTACCGGGTTG-3′). At maturity (R1, silks exposed), the plants were scored for pigmentation, and sheath tissue was dissected, snap frozen in liquid nitrogen, and stored at −80 °C.

### 2.5. FLAG-GAL4 Chromatin Immunoprecipitation and Mass-Spectrometry from Maize Sheath

For each biological replicate, 55–60 g of fresh sheath tissue was dissected from the mature plants and snap frozen in liquid nitrogen. Frozen tissue was then ground in liquid nitrogen by using a mortar and pestle. The tissue was further disrupted using a Waring Laboratory grade blender for 5 min on “low” in 300 mL of cross-linking nuclei isolation buffer 1 (10 mM Tris-HCl pH 8.0, 400 mM sucrose, 10 mM sodium-butyrate, 0.1 mM phenylmethylsuflonyl fluoride (PMSF), 5 mM β-mercaptoethanol, 0.33 µL/mL plant protease inhibitor cocktail (PIC, Sigma-Aldrich, St. Louis, MO, USA), 1% *v*/*v* formaldehyde). After homogenization and lysis, nuclear isolation was performed as previously described [32]. The chromatin was then fragmented with 60 units/mL of micrococcal nuclease (MNase; Worthington Biochemical, Lakewood, NJ, USA) to generate a distribution of nucleosomal bands that were mostly smaller than 1 kb but larger than 350 bp in length. Isolated soluble chromatin from two biological replicates (whole sheath tissue from individual plants) was used for each transgene combination, *UAS::b1TR; FLAG-GAL4*, *UAS::empty; FLAG-GAL4*, and—(no *UAS* transgene); *FLAG-GAL4*. Soluble chromatin from each biological replicate was diluted and incubated overnight at 4 °C with rotation with 4 µg of FLAG antibody (F3165, Sigma-Aldrich, St. Louis, MO, USA). An aliquot of soluble chromatin from the same biological replicate was incubated overnight at 4 °C with rotation with 20 µg of isotype control mouse IgG (I5381, Sigma-Aldrich, St. Louis, MO, USA) to identify common, non-specific proteins. Antibody–antigen complexes were captured using Dynabeads Protein G as per the manufacturer’s instructions (ThermoFisher, Bohemia, NY, USA). Beads were pooled and washed thrice with 750 µL of bead wash buffer 1 (0.01% *v*/*v* Tween-20, PIC in 1× PBS), thrice with bead wash buffer 2 (1× PBS, PIC), and twice with 100 mM ammonium bicarbonate before being transferred to a new 1.5 mL Protein Lo-Bind tube (Eppendorf, Hamburg, Germany) for a third wash. The supernatant was completely removed, and the beads were incubated in 20 µL of 10 ng/µL trypsin (ThermoFisher, Bohemia, NY, USA) in 100 mM ammonium bicarbonate. Digests were incubated for 4 h at 37 °C with occasional mixing by vortex. After digestion, the supernatant was collected off the magnetic beads and processed using C18-spin columns (ThermoFisher, Bohemia, NY, USA). The samples were lyophilized and the peptides were sequenced using the Thermo Q-Exactive LC-MS/MS (ThermoFisher, Bohemia, NY, USA) instrument as described in [33]. The aligned peptides were loaded into Scaffold ([34]; v4.10) for the visualization of identified proteins and statistical analysis. The candidate proteins selected for subsequent analysis had at least 1 peptide detected, a protein identification threshold set at 90.0%, and a peptide threshold at 95.0%. Randomized amino acid sequences of non-redundant proteins were used to determine the false discovery rate of the identified proteins across all of the samples (FDR = 1.2%).

### 2.6. ChIP-qPCR Enrichment Analysis of FLAG-GAL4 at UAS::b1TR

ChIP-qPCR was performed in triplicate using sheath tissue-derived soluble chromatin from *UAS::b1TR;FLAG-GAL4* that was incubated with FLAG (F3165, Sigma-Aldrich, St. Louis, MO, USA) and mouse IgG (I5381, Sigma-Aldrich, St. Louis, MO, USA) antibodies. After chromatin immunoprecipitation, Dynabeads beads were pooled and washed as described above. The ChIP-DNA was eluted and used as template in qPCR as previously described [32]. To differentiate enrichment at *UAS::b1TR* from the ChIP-qPCR signal derived from the endogenous *b1TR*, primers were used to target a region specific to the *UAS::b1TR* transgene that was not present elsewhere in the maize genome: KM1684 (5′-GGCGCTTTCTCATAGCTCAC-3′) and KM1687 (5′-AGTCGTGTCTTACCGGGTTG-3′).

### 2.7. Cloning for Yeast One-Hybrid Experiment

LacZ bait plasmid constructs (pVC4241-1) were cloned during a previous study [35] by the insertion of a *b1TR* fragment (base pairs 362–853 from one repeat unit and 1–361 from the next repeat unit) into a pLacZi reporter plasmid (Takara Bioscience (formerly Clontech), San Jose, CA, USA). Negative control bait plasmids (4231 *b1TR*-) were prepared through digestion of the LacZ bait with SalI and XhoI to remove the *b1TR* fragment followed by re-ligation. The positive control prey construct (KKY006/*b1TR*_pos) contains CBBP from pVC442 (now cpp2, Zm00001d031008) in pEXP-AD502 (Invitrogen, Waltham, MA, USA). The ZAG4 experimental prey construct (YGY001) was assembled via Gibson assembly with the NEBuilder HiFi DNA Assembly Cloning Kit (Ipswich, MA, USA). The CBBP cDNA in pVC442 was replaced by *Zag4* (Zm00001eb120710) cDNA using gene-specific primers “zag4 orf_fwd” (5′-aaaaagcaggcttgtcgaccATGATGACCGATCTGAGCTG-3′) and “zag4 orf_rev” (5′-ctgggtacgcgtgcggccgcTCATCCGAGTTGGAGGGTC-3′). The DDT4 prey plasmid (KKY004) was assembled from pVC442 by replacing CBBP cDNA with a DDT4 (Zm00001eb329910) cDNA sequence.

### 2.8. Yeast Transformation

Yeast (*Saccharomyces cerevisiae*) cells were transformed using the lithium acetate protocol [36]. An initial overnight culture in 5 mL of liquid YPD medium was grown at 30 °C on a wheel, followed by the inoculation of 15 mL of fresh YPD to an OD600 of between 0.2 and 0.3 and incubation at 30 °C with shaking at 200 rpm until OD600 reached 1. The yeast culture was harvested via centrifugation, washed with 1 mL ddH₂O, and resuspended in 1 mL 100 µM LiAc. The cells were pelleted at top speed for 15 s and the supernatant was removed. They were then resuspended in 250 µL 100 µM LiAc, vortexed, and aliquoted (50 µL per tube). The transformation mix was added, which consisted of 240 µL PEG (50% *w*/*v*), 69 uL ddH_2_O, 36 µL 1 M LiAc, 10 µL ssDNA, and 5 uL plasmid DNA. The mix was vortexed using a micromixer for 1 min, incubated at 30 °C for 30 min, and heat-shocked at 42 °C for 30 min. The cells were pelleted at top speed for 30 s, and the supernatant was removed and resuspended in 200 uL ddH₂O and was spread on plates for incubation at 30 °C for 3–4 days.

### 2.9. Quantitative X-Gal Assay for Measuring β-Galactosidase Activity

The quantitative X-gal assay for measuring β-galactosidase activity was performed following the method described by Trimborn et al. [37]. All of the transformed yeast cells were plated on SD Ura-/Tr- except for KKY002 (Supplemental Table S3), which was plated on SD Ura- media, and incubated at 30 °C for 48 h. After incubation, the yeast colonies were harvested and resuspended in Z-buffer, and OD600 was measured. A standardized yeast suspension (OD600 = 5.0) was prepared and subjected to three freeze–thaw cycles to permeabilize the cells. A total of 50 µL of the lysate was combined with a 330 uL X-gal master mix (agarose, PBS, β-mercaptoethanol, and X-gal) in a 96-well plate, and the reaction was monitored at room temperature until a blue color developed. Pictures of the reaction were taken at regular intervals, and image analysis was conducted using Fiji software with the ReadPlate 3.0 plugin. β-galactosidase activity was calculated based on the absorbance values.

### 2.10. Bioinformatic Analysis of Identified Proteins

PantherGO [38] was used to analyze gene-ontology terms associated with the identified proteins. Unique Uniprot identifiers of proteins based on their transgene association were used as input for PantherGO gene list enrichment analysis. Gene co-expression network analysis of *Ddt4* (GRMZM2G128176) was performed using the maize gene co-expression network with default settings [39] (https://www.bio.fsu.edu/mcginnislab/mcn/main_page.php (accessed on 10 July 2019). Protein–protein interaction network analysis of ZAG4 and DDT4 was performed using STRING with default settings [40] (https://string-db.org/ (accessed on 26 August 2019)). Interaction data was imported into Cytoscape v3.6.1 to generate network interaction plots. Jalview was used to visualize the pair-wise alignment of DDT4 and BPTF, and SMART protein domain graphics were used for visual reference of the protein domains.

## 3. Results

### 3.1. UAS::b1TR Triggers Silencing of B-I While UAS::Empty Does Not

**Figure 1:**
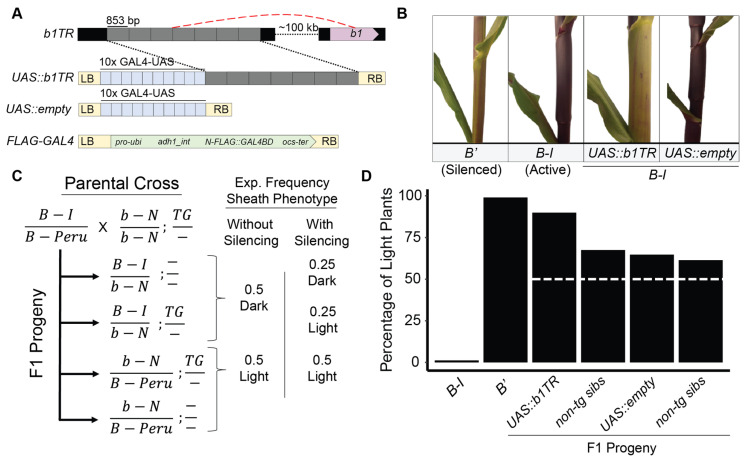
Pigmentation phenotypes of plants transgenic for *UAS::b1TR* and *UAS::empty* transgenes. (**A**) Schematic of the endogenous *b1TR* enhancer (red dashed line designates long-range chromatin loops that form with *b1*), and *UAS::b1TR*, *UAS::empty*, and *FLAG-GAL4* transgenes. The *UAS::b1TR* transgene encodes identical hepta repeats (grey boxes) cloned from *b1TR* fused to an adjacent 10× GAL4-binding upstream activation sequence array (UAS; blue boxes) providing unique binding sites for FLAG-GAL4. (**B**) Representative phenotypes of relevant *b1* genotypes (*B-I silenced (B’*) or *B-I active*) of non-transgenic plants and plants transgenic for either *UAS::b1TR* or *UAS::empty*. (**C**) Crossing scheme and expected sheath pigmentation with or without silencing for offspring segregating for a transgene (*UAS::b1TR* or *UAS::empty*) and *b1* alleles. (**D**) Percentage of light plants across all segregating families of transgenic and non-transgenic siblings and controls. White dashed line indicates expected proportion of light plants without *B-I* silencing.

To determine the effects of *UAS::b1TR* in the transgene-mediated silencing of *B-I*, we analyzed families of *B-I/b-N* plants segregating *UAS::b1TR* or *UAS::empty* transgenes for sheath pigmentation phenotypes (Figure 1A,B). Neutral alleles of *B* (referred to here as “*b-N*”) are recessive to *B-I* (active) and *B*′ (silenced), include only a truncated sequence at the *b1TR* locus, and do not enhance B1 expression or participate in paramutation (Coe 1959; [18,41]). Based on the genotypes and phenotypes of the parents used for these genetic experiments, lightly pigmented plants are only expected when plants are homozygous for *B*′ or *b-N*, *B’/b-N* heterozygous, when plants are transgenic for a sequence that induces silencing of the endogenous *B-I* gene, when spontaneous silencing of *b1TR* occurs in the plant, or when there is a genetic interaction with the Hi-II background of the transgenic parent (Figure 1C). Consistent with previously published work demonstrating the silencing of B1 by a *b1TR* transgene (Belele et al. [29]), *B-I* plants transgenic for *UAS::b1TR* were more often lightly pigmented (89.9%; n = 179; *p* = 0.1785) than with *UAS::empty* (64.7%; n = 167; *p* = 4.98 × 10^−6^) or any non-transgenic siblings segregating within families as determined in a blind, randomized field pigmentation scoring experiment (Figure 1D). Although this result does not exactly match the expectation of silencing in every transgenic plant, statistical analysis supports the hypothesis that the transgene induces the silencing of *B-I* in the analyzed progeny (Table 1). This suggests that *UAS::b1TR* interacts with *trans*-silencing machinery while *UAS::empty* likely does not. Although slightly higher-than-expected rates of light plants (expect 50% light based on genetic segregation, Figure 1C) in *UAS::empty* and non-transgenic siblings were observed, chi square analysis suggests that there is a high likelihood that this may have been caused by extraneous factors which may include insufficient sample size, genetic contamination, errors in genotyping, unpredicted genetic interactions between Hi-II and *B-I*, and/or observer error in color scoring in the field (Table 1).

### 3.2. Identification of Proteins Purified by FLAG-GAL4 ChIP-MS from Transgenic Plants

**Figure 2:**
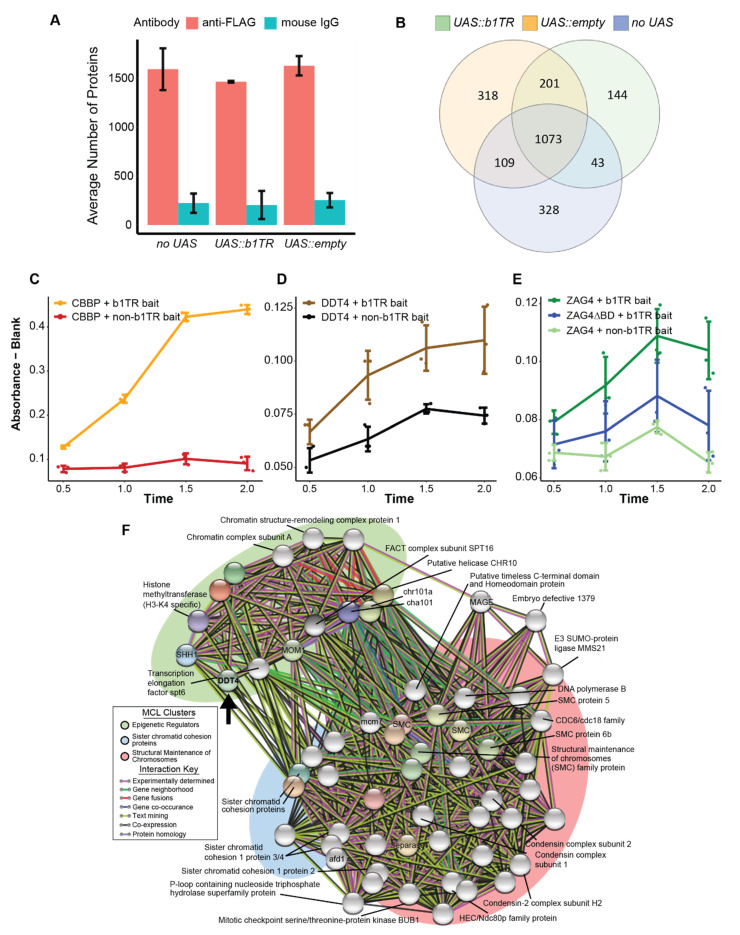
Results from UAS:transgene-targeted ChIP-MS, quantitative yeast one-hybrid assay results, and DDT4 interaction analysis (**A**) Average number of proteins identified by ChIP-MS for plants transgenic for the FLAG-GAL4 transgene without a UAS transgene (*no UAS*), or with a bait transgene (*UAS::b1TR* or *UAS::empty*). Error bars show the standard deviation for number of proteins between two biological replicates. (**B**) Distribution of identified proteins between bait transgenes from FLAG-GAL4 ChIP-MS experiments. (**C**) Beta-galactosidase expression was calculated based on absorbance values for *b1TR* or non-*b1TR* negative control bait and either CBBP b1TR binding protein prey (positive control), (**D**) DDT4 prey, or (**E**) ZAG4 full length or ZAG4ΔBD (without DNA binding domain) prey. (**F**) Expanded STRING protein–protein interaction network and MCL clustering with DDT4 primary interactors. The primary interactors of the DDT4-interacting protein set are shown as colored nodes. Secondary and tertiary interactions are shown as grey nodes. MCL clusters were manually annotated based on constituent proteins of each cluster. Nodes are connected by edges (lines) which are colored to indicate the interaction. The black arrow points to the location of DDT4 in the network.

To identify *UAS::b1TR*-interacting proteins, we performed anti-FLAG ChIP followed by mass-spectrometry using chromatin isolated from whole-plant sheath tissue from heterozygous *B-I* plants transgenic for *FLAG-GAL4* and *UAS::b1TR* (*B-I/b-N*; *UAS::b1TR/-*; *FLAG-GAL4/-*), *FLAG-GAL4* and *UAS::empty* (*B-I/b-N*; *UAS::empty/-*; *FLAG-GAL4/-*), or *FLAG-GAL4* without a UAS-containing transgene (*B-I/b-N*; *-/-*; *FLAG-GAL4/-*; *“no UAS”*). In the absence of a UAS-containing transgene, using ChIP to isolate FLAG-GAL4-associated DNA was not expected to capture locus-specific chromatin-interacting proteins, and these plants were used to identify non-specific proteins cross-linked to the FLAG-GAL4 protein or non-*b1TR* GAL4-associated DNA. After performing ChIP-MS, we found a total of 2432 non-redundant proteins across all samples (min. 1 peptide, 1.2% FDR). Proteins were identified with the antibody isotype control (mouse IgG) for all transgene combinations and these proteins represented less than 13% of all proteins detected (Figure 2A). To take a conservative approach and minimize false positives, we excluded all proteins shared between anti-FLAG and anti-IgG from further analysis, as they could not be confidently distinguished as true *UAS::b1TR*-interacting proteins. The remaining proteins present in anti-FLAG experiments were separated based on their transgene association, yielding 1461, 1701, and 1553 proteins from *UAS::b1TR*, *UAS::empty*, and *FLAG-GAL4* (“*no UAS*”), respectively, with 144 proteins uniquely identified by their association with *UAS::b1TR* (Figure 2B). To confirm that the protocol captured the FLAG-GAL4 fusion protein, we searched our obtained peptide spectra against the Swissprot reference database and identified the distribution of GAL4-associated peptides. As expected, GAL4 peptides (Uniprot accession P04386) were detected in all biological replicates using antibodies against FLAG while none were detected in any parallel mouse IgG ChIP reactions (Additional Dataset 2). In addition, we performed ChIP-qPCR targeting FLAG-GAL4 with antibodies against FLAG and mouse IgG. Consistent with enrichment by ChIP, we detected higher relative enrichment of the *UAS::b1TR* transgene sequence when using anti-FLAG compared to mouse IgG (Supplemental Figure S1A).

Although overlap among proteins was observed between transgene combinations, we identified distinct lists of proteins uniquely associated with *UAS::b1TR* and *UAS::empty*, providing candidates for further investigation (Figure 2B). We performed Gene Ontology (GO) analysis to identify terms associated with the captured proteins and found significantly enriched Biological Process GO terms (FDR < 0.05) for all anti-FLAG ChIP-MS experiments, with fewer significant terms found for experiments using mouse IgG as expected (Supplemental Figure S1B). Enriched Biological Process GO terms associated with unique *UAS::b1TR* proteins (FDR ≤ 0.02) include “regulation of gene expression”, “gene silencing”, “regulation of transcription by RNA Polymerase (Pol) II”, “nucleic acid metabolic process”, and “pigment biosynthetic process” (Supplemental Figure S1C). We also found that proteins annotated with the Molecular Function GO term “DNA-binding” were detected only in samples with UAS-containing transgenes, while none were detected in the FLAG-GAL4 alone (*no UAS*) control (Supplemental Figure S1D). In contrast, RNA-binding proteins showed both shared and distinct distributions across transgenes, with *UAS::b1TR*-associated proteins uniquely enriched for “double-stranded RNA binding” (Supplemental Figure S1E). These results suggest that our protein lists reflect the expected differences between UAS-containing chromatin and FLAG-GAL4 alone without a cognate UAS-binding site.

### 3.3. Several RNA-Binding Proteins Are Uniquely Associated with UAS::b1TR Chromatin

While proteins involved in *UAS::b1TR* regulation may act at multiple locations in the genome and may therefore be associated with the genomic location of any integrated transgene, the proteins unique to the *UAS::b1TR* are strong candidates for regulatory proteins acting specifically at the transgene locus. Gene ontology analysis was used to identify the predicted function of the 144 putative *UAS::b1TR*-interacting proteins (Figure 2B). Of the 95 out of 144 *UAS::b1TR*-associated proteins with annotated GO terms, 9 were classified as RNA-binding (Table 2). Several of these proteins contain features that may indicate a role in RNA processing. One is a predicted RNAse III domain-containing protein (Zm00001d012824) closely related to *Arabidopsis thaliana* NUCLEAR FUSION DEFECTIVE 2 (Nfd2), which functions in small RNA production through double-stranded RNA (dsRNA) binding. Two DEAD-box RNA helicases, DEAD-BOX RNA HELICASE 53 (ATRH53, Zm00001d041480), and EUKARYOTIC INITIATION FACTOR 4A-2 (EIF4A2, Zm00001d015251), were uniquely detected with *UAS::b1TR* and contain helicase domains that could facilitate dsRNA processing. Additionally, POLYADENYLATE-BINDING PROTEIN 4 (PAB4, Zm00001d019824) encodes four RNA RECOGNITION MOTIF (RRM) domains, a feature associated with proteins acting in co-transcriptional RNA processing. Other RNA-binding proteins, including the RRM-domain containing HETEROGENEOUS NUCLEAR RIBONUCLEOPROTEIN A3-LIKE PROTEIN 2 (HNRP3A, Zm00001d044469) and NUCLEAR TRANSPORT FACTOR 2 (NTF2, Zm00001d016528), were shared between the *UAS::b1TR* and *UAS::empty* transgenes (Table 2). While the functional relevance of these proteins in the context of *UAS::b1TR* remains unclear, their predicted activities are consistent with potential roles in co-transcriptional RNA processing.

### 3.4. Two Sequence-Specific DNA-Binding Proteins Were Uniquely Associated with UAS::b1TR Chromatin and Can Activate GAL Expression in Yeast One-Hybrid

In our analysis, two proteins encoding DNA binding domains were among the 144 found to be unique to *UAS::b1TR* (Table 3). AGAMOUS-LIKE MADS-BOX PROTEIN AGL5/ZEA AGAMOUS PROTEIN 4 (ZAG4; Zm00001eb120710) is a predicted sequence-specific transcription factor that may be associated with the RNA Pol II transcription of *b1TR*. ZAG4 is part of a highly-conserved gene family involved in floral homeotic gene expression [42] and is highly expressed in ear and husk leaves. We also identified DDT-TRANSCRIPTION FACTOR 4 (DDT4; Zm00001d022417) which is a 191 kDa protein of unknown function containing two AT-hook DNA binding domains, one DDT domain, and three PHD domains (Supplemental Figure S4A).

We performed *cis*-regulatory sequence analysis with a single 853 bp unit of the *b1TR* to determine if motifs suggestive of DDT4 or ZAG4 binding were present. While we did not detect putative ZAG4 binding sites, we detected several potential DDT4 binding sites throughout the *b1TR*, with most motifs positioned in the first 450 bp (Plant Pan 2.0, similarity score ≥ 0.91; Supplemental Table S4). AT-hook binding motifs were the most prevalent TF binding motif detected throughout the 853 bp unit, covering 18% of the sequence (Supplemental Figure S5). The predicted AT-hook binding motifs overlapped with experimentally-defined functional sequences within the *b1TR* including the CXC-DOMAIN CONTAINING B1-BINDING PROTEIN (CBBP) binding region [35], predicted yeast autonomous replicating sequences (ARS), and matrix attachment regions [14]. Genome-wide characterization of distal *cis*-elements in maize has identified several conserved distal element sequence motifs [43], one of which was detected at four sites within the 853 bp unit, with three out of four of these motifs overlapping with AT-hook binding motifs.

To assess whether the two DNA-binding proteins isolated from *UAS::b1TR* interact with *b1TR* in a heterologous system, we performed a quantitative yeast one-hybrid (Y1H) assay, which measures β-galactosidase activity as an indicator of transient interactions between proteins and target DNA sequences. In this assay, a plasmid containing the DNA sequence of interest (bait) and a reporter gene is transformed into yeast alongside another plasmid encoding the protein of interest (prey) fused to a GAL4 activation domain (AD). If the prey protein binds to the bait sequence, the GAL4 AD will drive reporter gene expression [37]. In this study, an 853 bp unit of the *b1TR* was used as bait alongside a LacZ reporter, with CBBP, DDT4, ZAG4, and ZAG4 with its DNA binding domain deleted (ZAG4ΔBD) as the prey (Supplemental Figure S2). As expected, the positive control prey CBBP, a protein shown to strongly activate GAL with *b1TR* bait [35], activated GAL expression when using an 853 bp unit of *b1TR* as bait (Figure 2C). Both DDT4 and ZAG4 activated GAL expression with *b1TR* bait, while no activation was observed in non-*b1TR* bait controls or when the ZAG4 DNA binding domain was deleted (Figure 2D,E). These results indicate that DDT4 and ZAG4 can activate GAL transcription in a *b1TR*-specific manner and provide supporting evidence for their association with *UAS::b1TR*-chromatin detected by ChIP-MS.

### 3.5. Predicted Interaction Networks of DDT4 and ZAG4 Suggest Chromatin-Associated Functions

We analyzed co-expression networks and protein–protein interaction predictions to explore the potential functions of *UAS::b1TR*-associated proteins, though additional experimental validation is needed to confirm their roles at this locus. STRING analysis [40] identified ZAG4 as a predicted interactor of AGAMOUS-LIKE MADS-BOX PROTEIN AGL6 (Zm00001d051465; Supplemental Figure S3A), a master regulator of floral development that recruits PRC2 for H3K27me3 deposition in Arabidopsis [44]. Expanding this network revealed additional predicted interactions with maize orthologs of FLOWERING LOCUS D (FLD, Zm00001d002684) and an RNA-RECOGNITION MOTIF (RRM) domain-containing protein (Zm00001d005281), the putative maize ortholog of FLOWERING CONTROL LOCUS A (FCA) (Supplemental Figure S3B). Although FCA was not detected in our ChIP-MS dataset, we detected several RRM-domain-containing proteins (PAB4, NTF2, and HRNPA3) associated with *UAS::b1TR* (Table 2).

DDT4 contains AT-hook DNA binding domains and PHD domains, which in other systems mediate chromatin interactions. DDT4 displays high amino acid similarity to human BROMODOMAIN PHD TRANSCRIPTION FACTOR (BPTF), which binds H3K4me3 and participates in chromatin remodeling (blastp score = 92.0, e-value = 9 × 10^−18^; Supplemental Figure S4B,C). Co-expression analysis using a maize gene expression network [39] identified associations with genes involved in chromatin modification, including SET DOMAIN GENE 127 (SDG127; GRMZM2G473138), the maize ortholog of ATSDG25/ATRX7 (Table 4), which regulates H3K4 demethylation in Arabidopsis [45]. DDT4 was also co-expressed with MEDIATOR OF RNA-POLYMERASE II TRANSCRIPTION SUBUNIT 14 (MED14; GRMZM2G446872; Table 4). Our proteomic analysis further detected another mediator complex subunit, HSP6, and the largest Pol II subunit, NRPB1, at *UAS::b1TR* (Table 3). STRING analysis predicted interactions between DDT4 and several major chromatin regulators, including H3K4-specific methyltransferases SET102 and ASHH1, as well as the ISWI ATPase CHR11, homologous to PICKLE/CHD3 (Table 5). DDT4 was also predicted to interact with CHR101 and CHR106 (Table 5), maize orthologs of DDM1, which maintain DNA methylation and epigenetic silencing [46,47,48,49]. In maize, DDM1 interacts with SHH2 and the RdDM machinery to maintain asymmetric DNA methylation and silence transposable elements [50].

An expanded STRING network incorporating DDT4 and its predicted primary interactors suggest DDT4 interacts broadly with chromatin regulators, chromosome cohesion proteins, and DNA replication factors (Supplemental Table S1). Markov clustering (MCL) (inflation parameter = 7) generated three clusters associated with epigenetic regulation, sister chromatid cohesion, and SMC-related proteins (Figure 2E). Overall, our identification of DDT4 and ZAG4 at *UAS::b1TR*, and their potential roles in transcriptional regulation, chromatin modification, and DNA replication may provide some insight into their involvement in the epigenetic silencing of *b1*; however, additional experiments are necessary to confirm their interactions and define their precise role at *b1TR*.

## 4. Discussion

Discovery-based proteomics experiments of complex mixtures can identify several thousand proteins, including many non-specific interactions, making interpretation challenging and requiring investigators to manually screen large datasets (reviewed in [51]). To attempt to overcome this, we used genetic controls (*UAS::empty* and *no UAS*) alongside *UAS::b1TR* to identify non-specific proteins that overlap between samples, reducing the effective search space for proteins of interest. The conservative filtering approach resulted in the identification of only two proteins with DNA binding domains, DDT4 and ZAG4, both of which interact with *b1TR* to drive GAL expression in yeast one-hybrid.

Consistent with our expectations of RNA Pol II transcription of *b1TR* and the transcriptional silencing associated with *UAS::b1TR* observed in this study, several of the proteins detected included RNA Pol II subunits, components of Mediator, and were involved in co-transcriptional RNA processing [15,22,29,52]. However, we did not detect CBBP, a CXC-domain containing zinc finger transcription factor that interacts with *b1TR* [35]. CBBP strongly activates GAL expression with *b1TR* bait in the yeast one-hybrid assay and is sufficient to induce silencing and paramutation when overexpressed [35]. The absence of CBBP in our ChIP-MS data could reflect inherent limitations in the technique or native tissue-specific expression of CBBP. CBBP (also known as CPP2; GRMZM2G015097) is expressed in seed and is low or not expressed in sheath tissue and may therefore not be interacting at *b1TR* in our samples (Supplemental Figure S6). This may also represent differences in the proteins involved in the establishment and maintenance stages of paramutation which occur during reproduction and vegetative growth, respectively [53]. Despite CBBP not being detected in our dataset, this does not compromise the overall conclusions of the study, which are based on factors active during the maintenance phase in vegetative tissue.

One of the few differences between *b1TR* in *B*′ versus *B-I* is the enrichment of H3K9me2 and H3K27me2 at silenced *b1TR* and H3K9ac at active *b1TR* [16,54]. Sequence-specific DNA binding proteins were expected to play an important role in *b1TR*-mediated silencing, enhancement, and paramutation of *b1* because of the local enrichment of transcription factor binding motifs present in the hepta-repeat relative to accessions with only one 853 bp copy, for example B73, which does not participate in paramutation or display enhancement of *b1* [13,14,29]. The requirement for repetitive motifs may be involved in higher-order oligomerization of transcription factors in three-dimensional chromatin organization [55], and may explain the lower GAL activation by DDT4 and ZAG4 relative to CBBP in our yeast one-hybrid assay, which did not include endogenous chromatin context, accessory factors, or seven tandem copies of *b1TR*.

The first PHD domain of DDT4 displayed high amino acid sequence conservation to the first PHD domain of human BPTF which binds H3K4me3 at actively transcribed loci ([56], reviewed in [57]). ZAG4 was predicted to interact with AGL6 and the maize ortholog of FLOWERING LOCUS D which, in Arabidopsis, recruits H3K4-specific demethylases and histone deacetylases HDAC5/6 to *Flowering locus C* (*FLC*) [58,59]. Despite the absence of the canonical MADS-box/SRF/MCM “CArG” (CC(A/T)6GG) recognition motif in *b1TR*, slight modifications to the consensus were tolerated with reduced affinity for AGAMOUS (AG) binding and this affinity varied depending on whether AG formed a homo or heterodimer with related AGs [60,61]. ZAG4 and DDT4 were predicted to interact with several maize H3K4 methyltransferases which may work to regulate Pol II transcription of *b1TR* and affect siRNA levels [52], leading to an increase in 24-nt siRNA biogenesis from *UAS::b1TR*, triggering *cis* and *trans* silencing of *b1TR*. This is exemplified by the copy number-dependent transition of 21-nt to 24-nt siRNA in de novo transposon silencing of the active *EVADE* retrotransposon by RdDM, which requires a threshold of ~40 copies before fully penetrant silencing is triggered [62,63].

Proteins related to co-transcriptional RNA processing and small RNA biogenesis were identified uniquely with *UAS::b1TR*, for example, the RNAse III domain containing protein NFD2 which interacted directly with 21-nt siRNA-producing DICER-LIKE 4 in *Arabidopsis thaliana* [64]. PAB4, HNRPA3, and NTF2 are RNA RECOGNITION MOTIF (RRM)-domain-containing proteins associated with *UAS::b1TR*. RRM domains are ubiquitous protein domains across eukaryotes that bind single stranded RNA and are associated with post-transcriptional gene regulation [65]. Although RRM-domain proteins were also detected in *UAS::empty* samples, we focused on the functional relevance of specific RNA-binding proteins uniquely associated with *UAS::b1TR* rather than on domain frequency alone. In Arabidopsis, the RRM-domain containing protein FLOWERING TIME CONTROL PROTEIN A (FCA) is required for the deposition of asymmetric DNA methylation at single/low copy repeat loci [66], and the maize FCA ortholog was predicted to interact with FLD, ZAG4 and AGL6. We also found HLN and PAI1 uniquely associated with *UAS::b1TR*. In humans, these proteins interact directly with CHD3 [67] and are involved in mRNA stability [68,69]. The *Drosophila melanogaster* orthologs of HLN and PAI1 and VIG and VIG2 are components of the RNA-induced silencing complex [70] that are involved in the inheritance of heterochromatin [71] and they have been shown to interact directly with the actively transcribed chromatin of the *Histone C* gene cluster [12]. These examples suggest that these RNA binding proteins may be important in co-transcriptional RNA processing and the recruitment of H3K4 demethylases and HDACs to establish transcriptional gene silencing of *b1TR*.

In this study, and to our knowledge for the first time in plants, we identified in vivo the chromatin-interacting proteins of a stably integrated transgene locus. The proteins that interact with *UAS::b1TR* chromatin may be responsible for the dramatic phenotypic differences between transcriptional enhancement and the silencing of *b1* between epi-alleles. Our approach has identified subunits of RNA Pol II, RNA-binding proteins, and chromatin-interacting proteins associated with *UAS::b1TR*, which may be involved in transgene-mediated *trans*-silencing of endogenous *b1TR*. In addition, we detected and confirmed sequence specificity through GAL activation of two unique *UAS::b1TR*-associated proteins, ZAG4 and DDT4. Subsequent genetic studies of these proteins will be critical to determine the mechanism for conferring the active versus silenced state of the *b1TR* in *B-I* and *B*′, respectively. In addition, the use of CRISPR-based ChIP-MS and proximity labeling approaches can improve our ability to identify and characterize the consortia of *trans* factors interacting with the *b1TR* and other *cis* regulatory loci, an important step towards efforts deciphering their effects on gene expression and inheritance.

## Figures and Tables

**Table 1:** Results of sheath pigmentation scoring for transgenic lines.

Genotype	No. Plant	Exp. Light	Obs. Light	Exp. Dark	Obs. Dark	*χ* ^2^	*p* Value
*UAS::b1TR/-*	179	179	161	0	18	1.81	0.1785
*UAS::empty/-*	167	167	108	0	59	20.84	4.98 × 10^−6^

Chi square test (*χ*^2^) was performed with phenotypic data for plants transgenic for *UAS::b1TR* or *UAS::empty* to estimate the degree of confidence for the null hypothesis of 1:0 light–dark plants, which would be expected if the transgene silenced *B-I*.

**Table 2:** Selected RNA-interacting proteins associated with the *UAS::b1TR*.

Protein Name	Gene ID	Accession	Predicted Function (GO)
Hyaluronan/mRNA binding family *	*Hln*	B4FR90	RNA-binding
Plasminogen activator inhibitor 1 RNA-binding protein *	*Pai1*	A0A1D6FLF3	RNA-binding
Polyadenylate-binding protein *	*Pab4*	A0A1D6I0G0	RNA-binding
Nuclear fusion defective 2 *	*Nfd2*	B6SGY5	production of siRNA in RNA-interference
DEAD-box ATP-dependent RNA helicase 53 *	*Atrh53*	A0A1D6MWD6	helicase activity
Eukaryotic initiation factor 4A-2 *	*Eif4a-2*	B4FBK3	helicase activity
Heterogeneous nuclear ribonucleoprotein A3-like protein 2 ^+^	*Hnrpa3*	A0A096QLU9	mRNA processing
Nuclear transport factor 2 ^+^	*Ntf2*	A0A1D6H8Q2	RNA binding
THO complex subunit 4 ^++^	*Tho4*	B6T346	RNA-binding
Ribonuclease (Tudor-SN1) ^++^	*Tsn1*	K7UVD7	gene silencing by RNA

* Proteins uniquely identified with *UAS::b1TR*. ^+^ Proteins shared between *UAS::b1TR* and *UAS::empty*. ^++^ Proteins shared between *UAS::b1TR, UAS::empty*, and *no-UAS*.

**Table 3:** Selected DNA-interacting and chromatin-interacting proteins associated with *UAS::b1TR*.

Protein Name	Gene ID	Accession	Predicted Function (GO)
DDT-transcription factor 4 *	*Ddt4*	A0A096S3Y8	metal ion binding
Agamous-like MADS-box protein AGL5 *	*Agl5/Zag4*	A0A1D6MHD4	DNA-binding transcription factor activity
DNA repair protein RAD23 ^+^	*Rad23*	B6TB61	nucleotide-excision repair
Putative AP2/EREBP transcription factor superfamily protein ^++^	*Ereb186*	A0A1D6FGH1	DNA-binding transcription factor activity
RNA_pol_Rpb1_2 domain-containing protein ^+^	*Nrpb1*	A0A1x7YI76	DNA-directed 5′-3′ RNA polymerase activity
Putative mediator of RNA polymerase II transcription subunit 37c ^++^	*Hsp6*	A0A1D6N7I4	ATPase activity
Actin-related protein 4 ^++^	*Arp4*	B4FNB4	regulation of transcription by RNA polymerase II
Regulator of chromosome condensation 2 ^++^	*Rcc2*	B4FTT2	chromatin binding
Nucleosome assembly protein 1 ^++^	*Nfa102*	A0A1D6FWR5	nucleosome assembly
Histone deacetylase complex subunit SAP18 ^++^	*Sap18*	B4FIB4	

* Proteins uniquely identified with *UAS::b1TR*. ^+^ Proteins shared between *UAS::b1TR* and *UAS::empty*. ^++^ Proteins shared between *UAS::b1TR*, *UAS::empty*, and *no-UAS*.

**Table 4:** *Ddt4* gene co-expression network analysis showing primary nodes.

**Gene Name**	**Gene ID**	**Predicted Function**	**E-Value**	**Score**
*Chr5*	GRMZM2G010342	chromatin assembly or disassembly	0	60.52
*Mybr85*	GRMZM2G089406	RNA splicing	3.00 × 10^−99^	31.28
*Fab1b*	GRMZM2G111208		0	55.19
*Btaf1*	GRMZM2G168096	nucleic acid binding	0	60.88
*Sin3-like 4*	GRMZM2G334457	regulation of transcription	0	51.44
*Ptm3*	GRMZM2G403562	regulation of transcription	3.00 × 10^−138^	33.65
*Med14*	GRMZM2G446872	positive regulation of transcription	0	53.2
*Sdg127*	GRMZM2G473138	histone lysine methylation	2.00 × 10^−98^	69.78

**Table 5:** STRING primary protein–protein interactions of maize DDT4.

**Protein Name**	**Gene ID**	**Co-Expression**	**Exp. Defined Interaction**	**Text Mining**	**Combined Score**
Histone methyltransferase (H3-K4 specific)	GRMZM2G352431	0	0.719	0.718	0.917
Uncharacterized protein	Zm00001d036601	0.832	0	0.209	0.861
Histone-lysine N-methyltransferase ASHH1	GRMZM2G147619	0.832	0	0.185	0.857
Sister chromatid cohesion protein	Zm00001d018657	0.832	0	0.185	0.857
Sister chromatid cohesion protein	Zm00001d007943	0.832	0	0.185	0.857
Chromatin complex subunit A101	GRMZM2G177165	0.059	0.657	0.458	0.809
Chromatin complex subunit A106	GRMZM2G071025	0.129	0.161	0.36	0.491
Putative helicase CHR10	GRMZM2G049168	0.129	0.161	0.36	0.491

## Data Availability

Seed for all transgenic and control lines are available upon request. Proteomic data from all experiments used for this analysis was exported from Scaffold and is available in Additional Dataset 1 and Additional Dataset 2 and data from yeast one-hybrid experiments is available in Additional Dataset 3.

## Supplementary Materials

The following supporting information can be downloaded at: https://www.mdpi.com/article/10.3390/plants14121863/s1, Figure S1: (A) Enrichment of UAS::*b1TR* chromatin fragments using anti-FLAG versus anti-mouse IgG detected by ChIP-qPCR. (B) Comparison of the number of enriched (FDR < 0.05) GO terms between transgenes and control ChIP-MS experiments using mouse IgG. (C) Shared and overlapping significantly enriched (FDR < 0.05) Biological Process GO terms between *UAS::b1TR* and *UAS::empty*. (D) Percentage of GO terms within the DNA Binding Proteins category unique to each transgene. Samples from plants that were not transgenic for a UAS-containing transgene (*no UAS*) did not capture DNA binding proteins. (E) Percentage of GO terms within the RNA Binding Proteins category unique to each transgene; Figure S2: (A) Preparation of transgenic yeast strains. (B) Quantitative yeast one-hybrid assay; Figure S3: (A) Predicted protein-protein interactions of ZAG4 showing primary interaction between ZAG4 and AGL6. (B) Expanded protein-protein interaction network using ZAG4 and AGL6 as anchor interactors. (C) Gene co-expression network analysis focused on DDT4. (D) STRING protein-protein interaction (PPI) network focused on DDT4; Figure S4: Visualization of domains and local homology of maize DDT4 and human BPTF. (A) SMART domain annotation of the maize DDT4 protein. (B) SMART domain annotation of the human BPTF protein. (C) Pair-wise amino acid sequence alignment between DDT4 and BPTF showing conservation between the DDT domains and first PHD domains made by Jalview; Figure S5: Annotation of *b1TR* sequence features; Figure S6: Gene expression atlas showing tissue specific expression of CBBP/CPP2 (GRMZM2G015097) mainly in seed and low/no expression in vegetative tissues; Table S1: Expanded protein-protein interactions involving DDT4 primary Protein-protein interactions; Table S2: 144 Proteins uniquely associated with *UAS::b1TR*; Table S3: Plasmid Constructs and Strains Used in the Yeast Transformation Experiment; Table S4: Transcription factor binding motif analysis of the b1TR.

## References

[1] Lis J.T. (2019). A 50 Year History of Technologies That Drove Discovery in Eukaryotic Transcription Regulation. Nat. Struct. Mol. Biol..

[2] Wang M.M., Reed R.R. (1993). Molecular Cloning of the Olfactory Neuronal Transcription Factor Olf-1 by Genetic Selection in Yeast. Nature.

[3] Jutras B.L., Verma A., Stevenson B. (2012). Identification of Novel DNA-Binding Proteins Using DNA-Affinity Chromatography/Pull Down. Curr. Protoc. Microbiol..

[4] Déjardin J., Kingston R.E. (2009). Purification of Proteins Associated with Specific Genomic Loci. Cell.

[5] Byrum S.D., Raman A., Taverna S.D., Tackett A.J. (2012). ChAP-MS: A Method for Identification of Proteins and Histone Posttranslational Modifications at a Single Genomic Locus. Cell Rep..

[6] Pourfarzad F., Aghajanirefah A., de Boer E., Ten Have S., Bryn van Dijk T., Kheradmandkia S., Stadhouders R., Thongjuea S., Soler E., Gillemans N. (2013). Locus-Specific Proteomics by TChP: Targeted Chromatin Purification. Cell Rep..

[7] Gao X.D., Tu L.-C., Mir A., Rodriguez T., Ding Y., Leszyk J., Dekker J., Shaffer S.A., Zhu L.J., Wolfe S.A. (2018). C-BERST: Defining Subnuclear Proteomic Landscapes at Genomic Elements with dCas9–APEX2. Nat. Methods.

[8] Fujita T., Fujii H. (2013). Efficient Isolation of Specific Genomic Regions and Identification of Associated Proteins by Engineered DNA-Binding Molecule-Mediated Chromatin Immunoprecipitation (enChIP) Using CRISPR. Biochem. Biophys. Res. Commun..

[9] Liu X., Zhang Y., Chen Y., Li M., Zhou F., Li K., Cao H., Ni M., Liu Y., Gu Z. (2017). In Situ Capture of Chromatin Interactions by Biotinylated dCas9. Cell.

[10] Fujita T., Yuno M., Fujii H. (2018). enChIP Systems Using Different CRISPR Orthologues and Epitope Tags. BMC Res. Notes.

[11] Myers S.A., Wright J., Peckner R., Kalish B.T., Zhang F., Carr S.A. (2018). Discovery of Proteins Associated with a Predefined Genomic Locus via dCas9–APEX-Mediated Proximity Labeling. Nat. Methods.

[12] Tsui C., Inouye C., Levy M., Lu A., Florens L., Washburn M.P., Tjian R. (2018). dCas9-Targeted Locus-SPECIFIC protein Isolation Method Identifies Histone Gene Regulators. Proc. Natl. Acad. Sci. USA.

[13] Stam M., Belele C., Dorweiler J.E., Chandler V.L. (2002). Differential Chromatin Structure Within a Tandem Array 100 kb Upstream of the Maize b1 Locus Is Associated with Paramutation. Genes Dev..

[14] Stam M., Belele C., Ramakrishna W., Dorweiler J.E., Bennetzen J.L., Chandler V.L. (2002). The Regulatory Regions Required for B′ Paramutation and Expression Are Located Far Upstream of the Maize b1 Transcribed Sequences. Genetics.

[15] Louwers M., Bader R., Haring M., van Driel R., de Laat W., Stam M. (2009). Tissue- and Expression Level–Specific Chromatin Looping at Maize b1 Epialleles. Plant Cell.

[16] Haring M., Bader R., Louwers M., Schwabe A., van Driel R., Stam M. (2010). The Role of DNA Methylation, Nucleosome Occupancy and Histone Modifications in Paramutation. Plant J..

[17] Chandler V.L. (2010). Paramutation’s Properties and Puzzles. Science.

[18] Coe E.H. (1959). A Regular And Continuing Conversion-Type Phenomenon At The B Locus In Maize. Proc. Natl. Acad. Sci. USA.

[19] Patterson G.I., Thorpe C.J., Chandler V.L. (1993). Paramutation, an Allelic Interaction, Is Associated with a Stable and Heritable Reduction of Transcription of the Maize B Regulatory Gene. Genetics.

[20] Hollick J.B., Chandler V.L. (2001). Genetic Factors Required to Maintain Repression of a Paramutagenic Maize pl1 Allele. Genetics.

[21] Alleman M., Sidorenko L., McGinnis K., Seshadri V., Dorweiler J.E., White J., Sikkink K., Chandler V.L. (2006). An RNA-Dependent RNA Polymerase Is Required for Paramutation in Maize. Nature.

[22] Sloan A.E., Sidorenko L., McGinnis K.M. (2014). Diverse Gene-Silencing Mechanisms with Distinct Requirements for RNA Polymerase Subunits in *Zea mays*. Genetics.

[23] Hollick J.B., Kermicle J.L., Parkinson S.E. (2005). Rmr6 Maintains Meiotic Inheritance of Paramutant States in *Zea mays*. Genetics.

[24] Barbour J.-E.R., Liao I.T., Stonaker J.L., Lim J.P., Lee C.C., Parkinson S.E., Kermicle J., Simon S.A., Meyers B.C., Williams-Carrier R. (2012). Required to Maintain Repression2 is a Novel Protein that Facilitates Locus-Specific Paramutation in Maize. Plant Cell.

[25] Stonaker J.L., Lim J.P., ErhardErhard K.F., Hollick J.B. (2009). Diversity of Pol IV Function Is Defined by Mutations at the Maize rmr7 Locus. PLoS Genet..

[26] Deans N.C., Giacopelli B.J., Hollick J.B. (2020). Locus-Specific Paramutation in *Zea mays* Is Maintained by a PICKLE-like Chromodomain Helicase DNA-Binding 3 Protein Controlling Development and Male Gametophyte Function. PLoS Genet..

[27] Chandler V., Alleman M. (2008). Paramutation: Epigenetic Instructions Passed Across Generations. Genetics.

[28] Hollick J.B. (2017). Paramutation and Related Phenomena in Diverse Species. Nat. Rev. Genet..

[29] Belele C.L., Sidorenko L., Stam M., Bader R., Arteaga-Vazquez M.A., Chandler V.L. (2013). Specific Tandem Repeats Are Sufficient for Paramutation-Induced Trans-Generational Silencing. PLoS Genet..

[30] Brand A.H., Perrimon N. (1993). Targeted Gene Expression as a Means of Altering Cell Fates and Generating Dominant Phenotypes. Development.

[31] Armstrong C.L., Green C.E. (1985). Establishment and Maintenance of Friable, Embryogenic Maize Callus and the Involvement of L-proline. Planta.

[32] Haring M., Offermann S., Danker T., Horst I., Peterhansel C., Stam M. (2007). Chromatin Immunoprecipitation: Optimization, Quantitative Analysis and Data Normalization. Plant Methods.

[33] Gumber H.K., McKenna J.F., Estrada A.L., Tolmie A.F., Graumann K., Bass H.W. (2019). Identification and Characterization of Genes Encoding the Nuclear Envelope LINC Complex in the Monocot Species *Zea mays*. J. Cell Sci..

[34] Searle B.C. (2010). Scaffold: A Bioinformatic Tool for Validating MS/MS-Based Proteomic Studies. Proteomics.

[35] Brzeska K., Brzeski J., Smith J., Chandler V.L. (2010). Transgenic expression of CBBP, a CXC domain protein, establishes paramutation in maize. Proc. Natl. Acad. Sci. USA.

[36] Agatep R., Kirkpatrick R.D., Parchaliuk D.L., Woods R.A., Gietz R.D. (1998). Transformation of *Saccharomyces cerevisiae* by the Lithium Acetate/Single-Stranded Carrier DNA/Polyethylene Glycol Protocol. Tech. Tips Online.

[37] Trimborn L., Hoecker U., Ponnu J. (2022). A Simple Quantitative Assay for Measuring β-Galactosidase Activity Using X-Gal in Yeast-Based Interaction Analyses. Curr. Protoc..

[38] Mi H., Muruganujan A., Ebert D., Huang X., Thomas P.D. (2019). PANTHER Version 14: More Genomes, a New PANTHER GO-Slim and Improvements in Enrichment Analysis Tools. Nucleic Acids Res..

[39] Huang J., Zheng J., Yuan H., McGinnis K. (2018). Distinct Tissue-Specific Transcriptional Regulation Revealed by Gene Regulatory Networks in Maize. BMC Plant Biol..

[40] Szklarczyk D., Gable A.L., Lyon D., Junge A., Wyder S., Huerta-Cepas J., Simonovic M., Doncheva N.T., Morris J.H., Bork P. (2019). STRING v11: Protein–Protein Association Networks with Increased Coverage, Supporting Functional Discovery in Genome-Wide Experimental Datasets. Nucleic Acids Res..

[41] Chandler V.L., Radicella J.P., Robbins T.P., Chen J., Turks D. (1989). Two Regulatory Genes of the Maize Anthocyanin Pathway Are Homologous: Isolation of B Utilizing R Genomic Sequences. Plant Cell.

[42] Mena M., Mandel M.A., Lerner D.R., Yanofsky M.F., Schmidt R.J. (1995). A Characterization of the MADS-Box Gene Family in Maize. Plant J..

[43] Peng Y., Xiong D., Zhao L., Ouyang W., Wang S., Sun J., Zhang Q., Guan P., Xie L., Li W. (2019). Chromatin Interaction Maps Reveal Genetic Regulation for Quantitative Traits in Maize. Nat. Commun..

[44] Liu X., Kim Y.J., Müller R., Yumul R.E., Liu C., Pan Y., Cao X., Goodrich J., Chen X. (2011). AGAMOUS Terminates Floral Stem Cell Maintenance in Arabidopsis by Directly Repressing WUSCHEL through Recruitment of Polycomb Group Proteins. Plant Cell.

[45] Berr A., Xu L., Gao J., Cognat V., Steinmetz A., Dong A., Shen W.-H. (2009). SET DOMAIN GROUP25 Encodes a Histone Methyltransferase and Is Involved in FLOWERING LOCUS C Activation and Repression of Flowering. Plant Physiol..

[46] Gendrel A.-V., Lippman Z., Yordan C., Colot V., Martienssen R.A. (2002). Dependence of Heterochromatic Histone H3 Methylation Patterns on the Arabidopsis Gene DDM1. Science.

[47] Zemach A., Kim M.Y., Hsieh P.-H., Coleman-Derr D., Eshed-Williams L., Thao K., Harmer S.L., Zilberman D. (2013). The Arabidopsis Nucleosome Remodeler DDM1 Allows DNA Methyltransferases to Access H1-Containing Heterochromatin. Cell.

[48] Lee S.C., Adams D.W., Ipsaro J.J., Cahn J., Lynn J., Kim H.-S., Berube B., Major V., Calarco J.P., LeBlanc C. (2023). Chromatin Remodeling of Histone H3 Variants by DDM1 Underlies Epigenetic Inheritance of DNA Methylation. Cell.

[49] Shimada A., Cahn J., Ernst E., Lynn J., Grimanelli D., Henderson I., Kakutani T., Martienssen R.A. (2024). Retrotransposon Addiction Promotes Centromere Function via Epigenetically Activated Small RNAs. Nat. Plants.

[50] Long J.C., Xia A.A., Liu J.H., Jing J.L., Wang Y.Z., Qi C.Y., He Y. (2019). Decrease in DNA methylation 1 (DDM1) Is Required for the Formation of mCHH Islands in Maize. J. Integr. Plant Biol..

[51] Zubarev R.A. (2013). The Challenge of the Proteome Dynamic Range and Its Implications for In-Depth Proteomics. Proteomics.

[52] Arteaga-Vazquez M., Sidorenko L., Rabanal F.A., Shrivistava R., Nobuta K., Green P.J., Meyers B.C., Chandler V.L. (2010). RNA-Mediated Trans-Communication Can Establish Paramutation at the b1 Locus in Maize. Proc. Natl. Acad. Sci. USA.

[53] Dorweiler J.E., Carey C.C., Kubo K.M., Hollick J.B., Kermicle J.L., Chandler V.L. (2000). Mediator of Paramutation1 Is Required for Establishment and Maintenance of Paramutation at Multiple Maize Loci. Plant Cell.

[54] Hövel I., Bader R., Louwers M., Haring M., Peek K., Gent J.I., Stam M. (2024). RNA-Directed DNA Methylation Mutants Reduce Histone Methylation at the Paramutated Maize Booster1 Enhancer. Plant Physiol..

[55] Kim S., Shendure J. (2019). Mechanisms of Interplay Between Transcription Factors and the 3D Genome. Mol. Cell.

[56] Wysocka J., Swigut T., Xiao H., Milne T.A., Kwon S.Y., Landry J., Kauer M., Tackett A.J., Chait B.T., Badenhorst P. (2006). A PHD Finger of NURF Couples Histone H3 Lysine 4 Trimethylation with Chromatin Remodelling. Nature.

[57] Alkhatib S.G., Landry J.W. (2011). The Nucleosome Remodeling Factor. FEBS Lett..

[58] Yu C.-W., Liu X., Luo M., Chen C., Lin X., Tian G., Lu Q., Cui Y., Wu K. (2011). HISTONE DEACETYLASE6 Interacts with FLOWERING LOCUS D and Regulates Flowering in Arabidopsis. Plant Physiol..

[59] Luo M., Tai R., Yu C.-W., Yang S., Chen C.-Y., Lin W.-D., Schmidt W., Wu K. (2015). Regulation of Flowering Time by the Histone Deacetylase HDA5 in Arabidopsis. Plant J..

[60] Huang H., Mizukami Y., Hu Y., Ma H. (1993). Isolation and Characterization of the Binding Sequences for the Product of the Arabidopsis Floral Homeotic Gene AGAMOUS. Nucleic Acids Res..

[61] Shiraishi H., Okada K., Shimura Y. (1993). Nucleotide Sequences Recognized by the AGAMOUS MADS Domain of Arabidopsis Thaliana in Vitro. Plant J..

[62] Marí-Ordóñez A., Marchais A., Etcheverry M., Martin A., Colot V., Voinnet O. (2013). Reconstructing de Novo Silencing of an Active Plant Retrotransposon. Nat. Genet..

[63] Trasser M., Bohl-Viallefond G., Barragán-Borrero V., Diezma-Navas L., Loncsek L., Nordborg M., Marí-Ordóñez A. (2024). PTGS Is Dispensable for the Initiation of Epigenetic Silencing of an ACTIVE transposon in Arabidopsis. bioRxiv.

[64] Montavon T., Kwon Y., Zimmermann A., Hammann P., Vincent T., Cognat V., Bergdoll M., Michel F., Dunoyer P. (2018). Characterization of DCL4 Missense Alleles Provides Insights into Its Ability to Process Distinct Classes of dsRNA Substrates. Plant J..

[65] Maris C., Dominguez C., Allain F.H.-T. (2005). The RNA Recognition Motif, a Plastic RNA-Binding Platform to Regulate Post-Transcriptional Gene Expression. FEBS J..

[66] Bäurle I., Smith L., Baulcombe D.C., Dean C. (2007). Widespread Role for the Flowering-Time Regulators FCA and FPA in RNA-Mediated Chromatin Silencing. Science.

[67] Lemos T.A., Passos D.O., Nery F.C., Kobarg J. (2003). Characterization of a New Family of Proteins That Interact with the C-Terminal region of the Chromatin-Remodeling Factor CHD-31. FEBS Lett..

[68] Kobarg C.B., Kobarg J., Crosara-Alberto D.P., Theizen T.H., Franchini K.G. (2005). MEF2C DNA-Binding Activity Is Inhibited Through Its Interaction with the Regulatory Protein Ki-1/57. FEBS Lett..

[69] Bressan G.C., Quaresma A.J.C., Moraes E.C., Manfiolli A.O., Passos D.O., Gomes M.D., Kobarg J. (2009). Functional Association of Human Ki-1/57 with Pre-mRNA SPLICING events. FEBS J..

[70] Gracheva E., Dus M., Elgin S.C.R. (2009). Drosophila RISC Component VIG and Its Homolog Vig2 Impact Heterochromatin Formation. PLoS ONE.

[71] Schneiderman J.I., Goldstein S., Ahmad K. (2010). Perturbation Analysis of Heterochromatin-Mediated Gene Silencing and Somatic Inheritance. PLoS Genet..

