# Supplementary material for: Identification of Proteins Associated with Stably Integrated Maize b1 Tandem Repeat Transgene Chromatin
Source: Plants (Basel). 2025 Jun 17;14(12):1863. doi: 10.3390/plants14121863 (PMC12196572; doi:10.3390/plants14121863)
Supplement: Supplementary file 1 [file plants-14-01863-s001.zip › plants-3611153-supplementary.pdf]

SUPPLEMENTAL MATERIALS

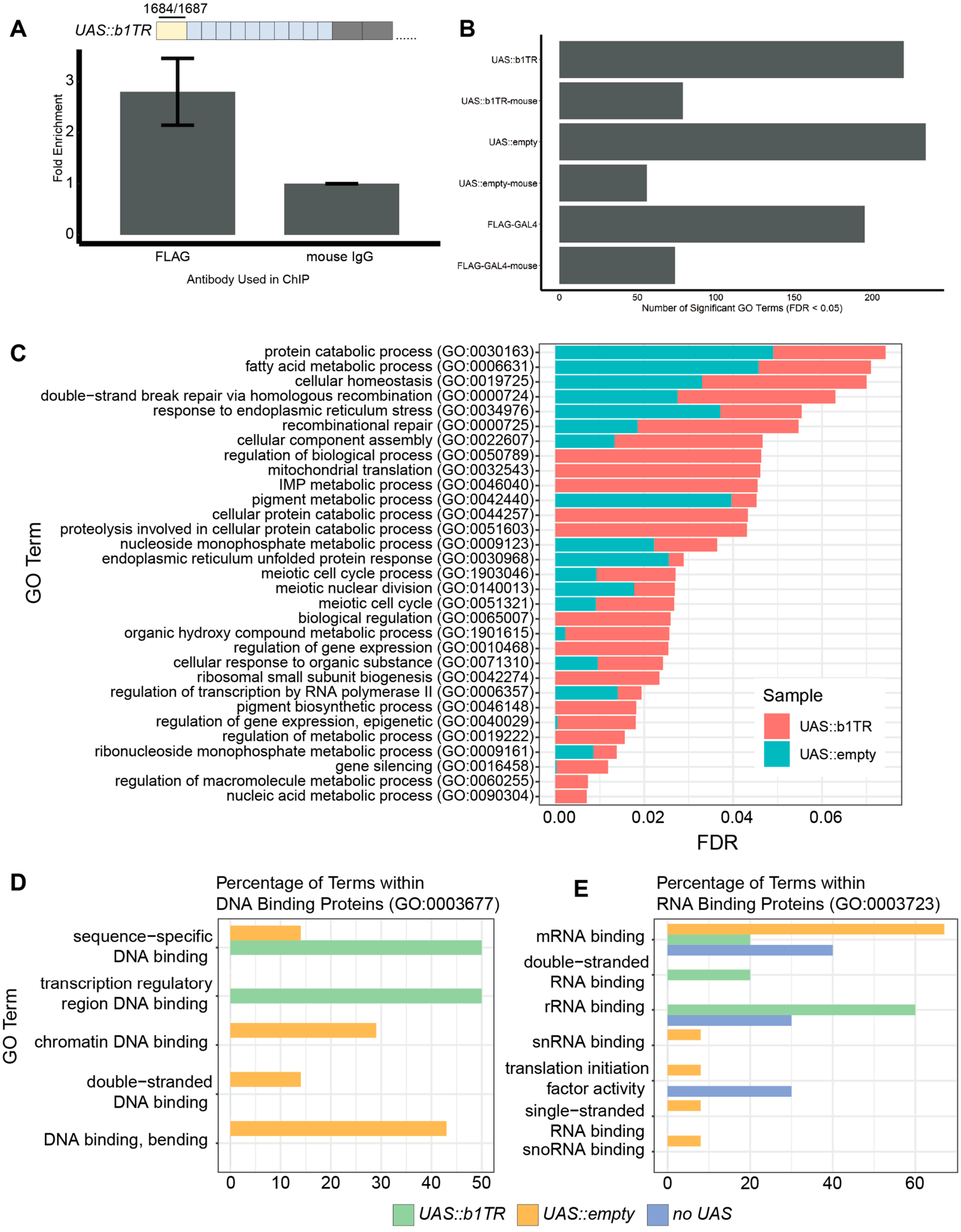

Supplemental Figure S1. (**A**) Enrichment of UAS::*b1TR* chromatin fragments using anti-FLAG versus anti-mouse IgG detected by ChIP-qPCR. (**B**) Comparison of the number of enriched (FDR < 0.05) GO terms between transgenes and control ChIP-MS experiments using mouse IgG. (**C**) Shared and overlapping significantly enriched (FDR < 0.05) Biological Process GO terms between *UAS::b1TR* and *UAS::empty*. (**D**) Percentage of GO terms within the DNA Binding Proteins category unique to each transgene. Samples from plants that were not transgenic for a UAS-containing transgene (*no UAS)* did not capture DNA binding proteins. (**E**) Percentage of GO terms within the RNA Binding Proteins category unique to each transgene.

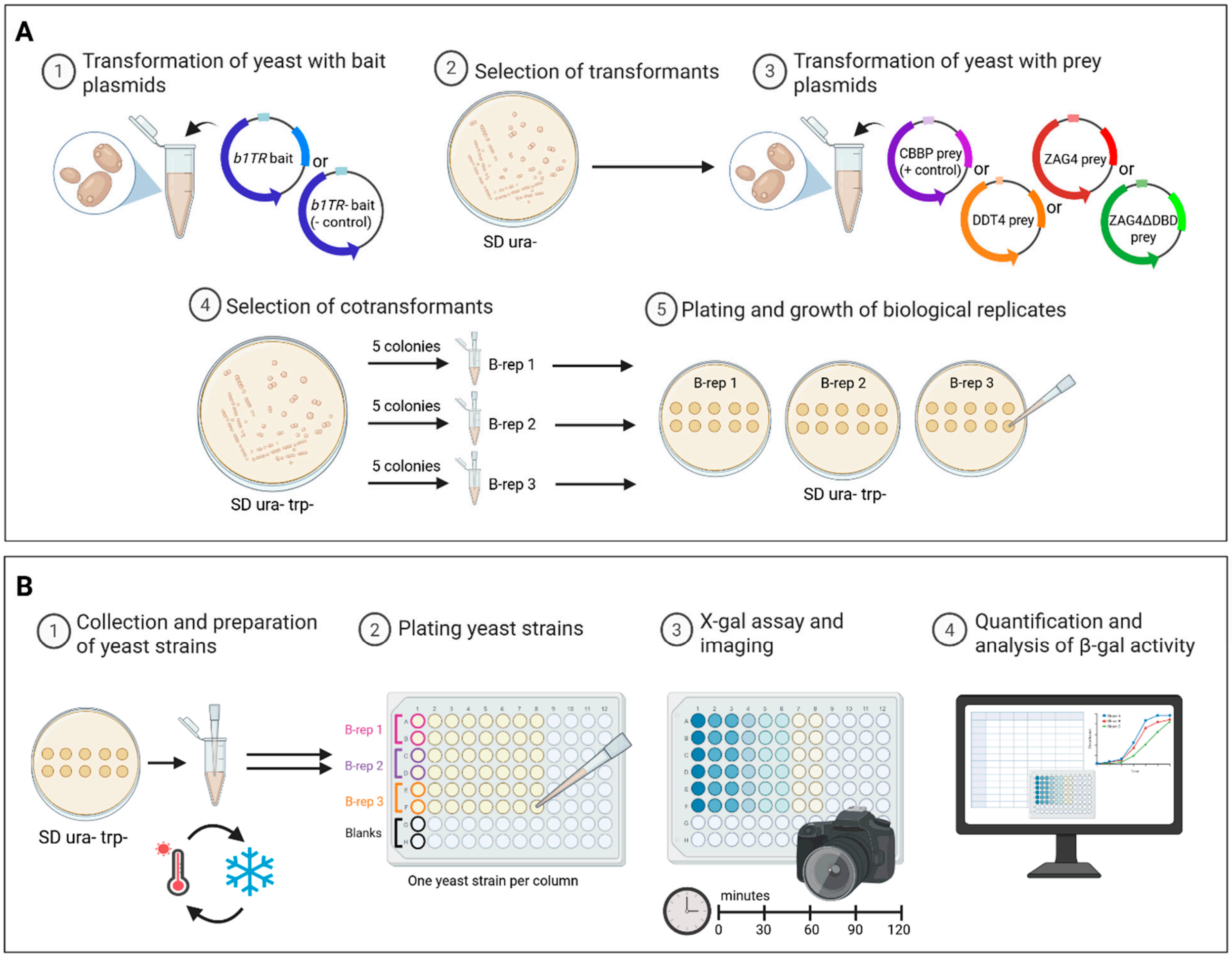

Supplemental Figure S2. (**A**) Preparation of transgenic yeast strains. Competent yeast cells were transformed first with one of two bait plasmids, the *b1TR* bait or the negative control *b1TR-* bait. Transformant colonies were selected for with SD ura- media and subsequently transformed with one of four prey plasmids: CBBP (positive control), DDT4, ZAG4, or ZAG4ΔDBD. Co-transformants were selected for with SD ura- trp- media. A total of 15 colonies from each strain were selected and pooled in groups of 5 colonies per biological replicate, then plated on SD ura- trp- media for growth. (**B**) Quantitative yeast one-hybrid assay. Yeast was harvested from SD ura- trp- plates for each biological replicate and resuspended in Z-buffer before being subjected to three freeze-thaw cycles. Each yeast strain was pipetted into wells of the same 96-well plate, with each biological replicate being divided among two wells for technical replication. An additional two wells were filled only with Z-buffer to serve as blanks. After the addition of an X-gal buffer to each well, the reaction was monitored for 120 minutes with imaging every 30 minutes. β-galactosidase activity was then calculated from the absorbances and compared across samples.

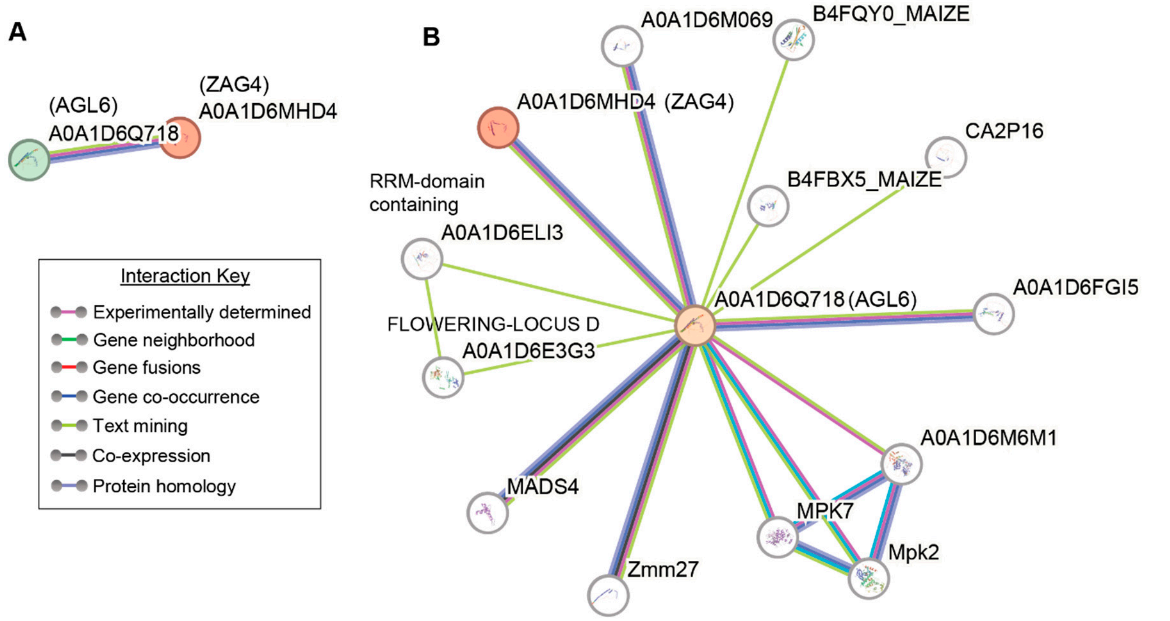

node	domain_summary_url
A0A1D6E3G3	Flowering locus D.
A0A1D6ELI3	RNA binding.
A0A1D6FGI5	AGAMOUS-like protein.
A0A1D6M069	ZAG1 protein.
A0A1D6M6M1	Serine/threonine-protein kinase STN8 chloroplastic.
A0A1D6MHD4	Agamous-like MADS-box protein AGL5.
A0A1D6Q718	Agamous-like MADS-box protein AGL6.
B4FBX5_MAIZE	DNA excision repair protein ERCC-1.
B4FQY0_MAIZE	Pathogenesis-related protein PR-1; Belongs to the CRISP family.
CA2P16	Nuclear transcription factor Y subunit A-1.
MADS4	Developmental protein SEPALLATA 2.
MPK7	Mitogen-activated protein kinase; Belongs to the protein kinase superfamily. Ser/Thr protein kinase family. MAP kinase subfamily.
Mpk2	Mitogen-activated protein kinase; Belongs to the protein kinase superfamily. Ser/Thr protein kinase family. MAP kinase subfamily.
Zmm27	MADS27.

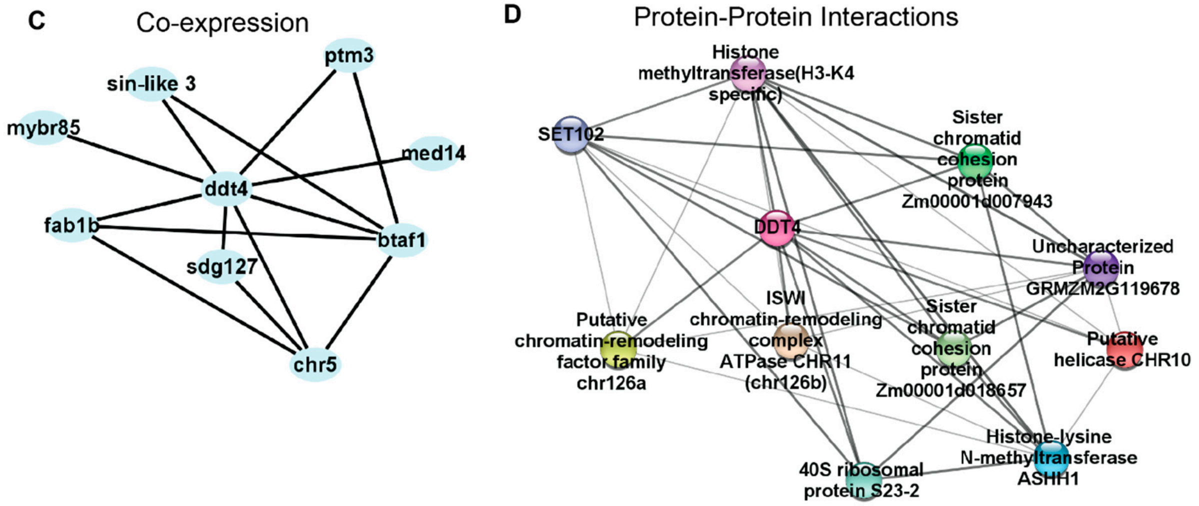

Supplemental Figure S3. (**A**) Predicted protein-protein interactions of ZAG4 showing primary interaction between ZAG4 and AGL6. (**B**) Expanded protein-protein interaction network using ZAG4 and AGL6 as anchor interactors. FLC regulators shown middle left contain RRM-domain containing protein FCA, and FLOWERING-LOCUS D. Table showing descriptive domain summaries for each UNIPROT identifier in STRING PPI network. (**C**) Gene co-expression network analysis focused on DDT4. Cyan ellipses indicate nodes and lines indicate edges. Gene names are inferred from *Arabidopsis thaliana* closest homologs if maize gene models are unnamed. (**D**) STRING protein-protein interaction (PPI) network focused on DDT4. Colored nodes represent primary interactors and black lines represent primary edges. Grey lines represent secondary edges (relationships between all nodes).

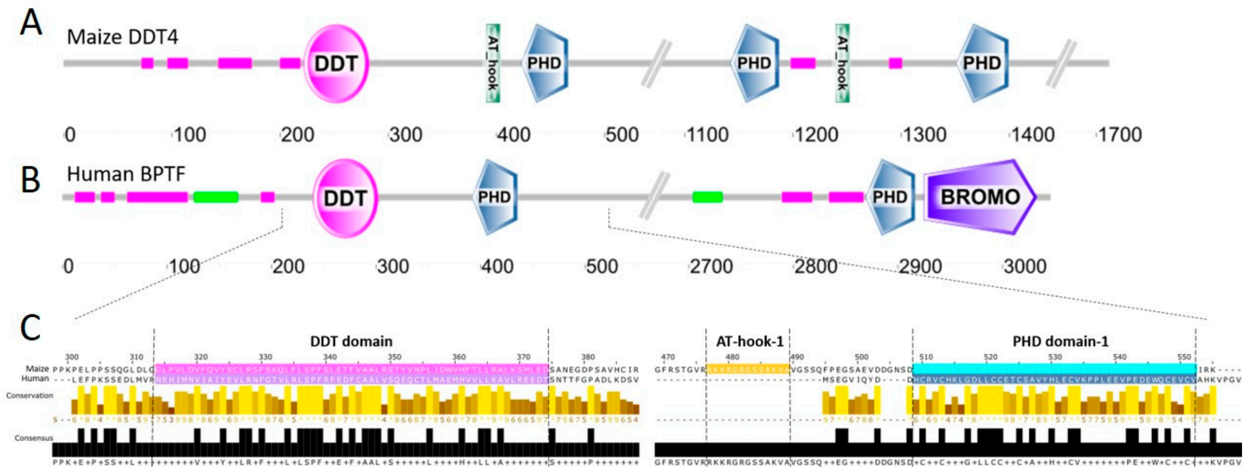

Supplemental Figure S4. Visualization of domains and local homology of maize DDT4 and human BPTF. (**A**) SMART domain annotation of the maize DDT4 protein. Position of domains (shapes) are indicated by amino acid number below each figure. Pink rectangles indicate predicted intrinsically disordered domains. Maize DDT4 has two AT hook domains flanked by PHD domains suggesting that it can interact with genetic and epigenetic cues. (**B**) SMART domain annotation of the human BPTF protein. Green rectangles indicate predicted coiled-coil motifs. (**C**) Pair-wise amino acid sequence alignment between DDT4 and BPTF showing conservation between the DDT domains and first PHD domains made by Jalview. DDT4 is the closest relative to BPTF in the maize non-redundant protein sequence database based on amino acids 200–550.

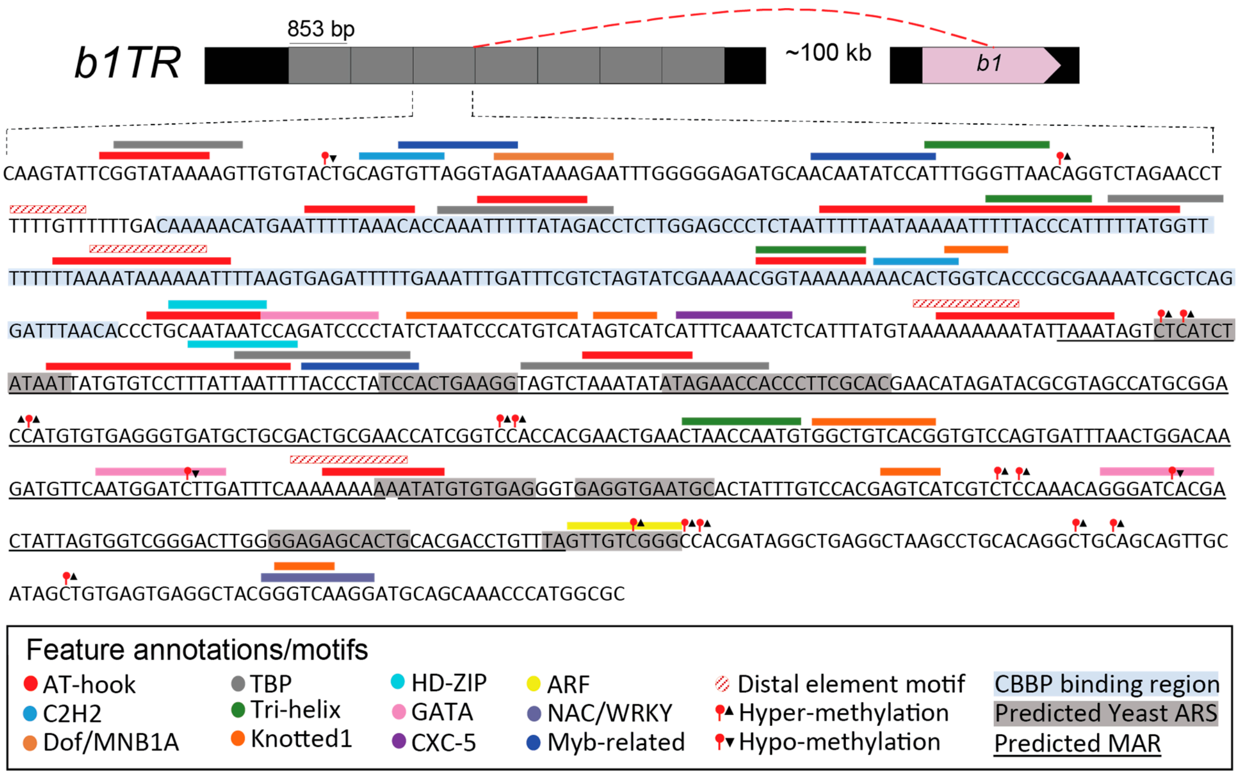

Supplemental Figure S5. Annotation of *b1TR* sequence features. Sequence analysis was used on a single copy of the 853 bp *b1TR* (TFBS cutoff, similarity ≥ 91%) to identify potential promoter elements, including predicted transcription factor binding sites. Predicted transcription factor binding sites are indicated by colored bars above the + strand sequence. Elements identified in other studies are also indicated and include a distal element motif (Peng et al., 2019 [42]), hyper and hypo methylation sites for b1 epialleles (Haring et al., 2010 [16]), CBBP binding region (Brzeska et al., 2010 [35]) and predicted matrix attachment regions (MAR) and yeast autonomously replicating sequence (ARS) (Stam et al., 2002 [13,14]).

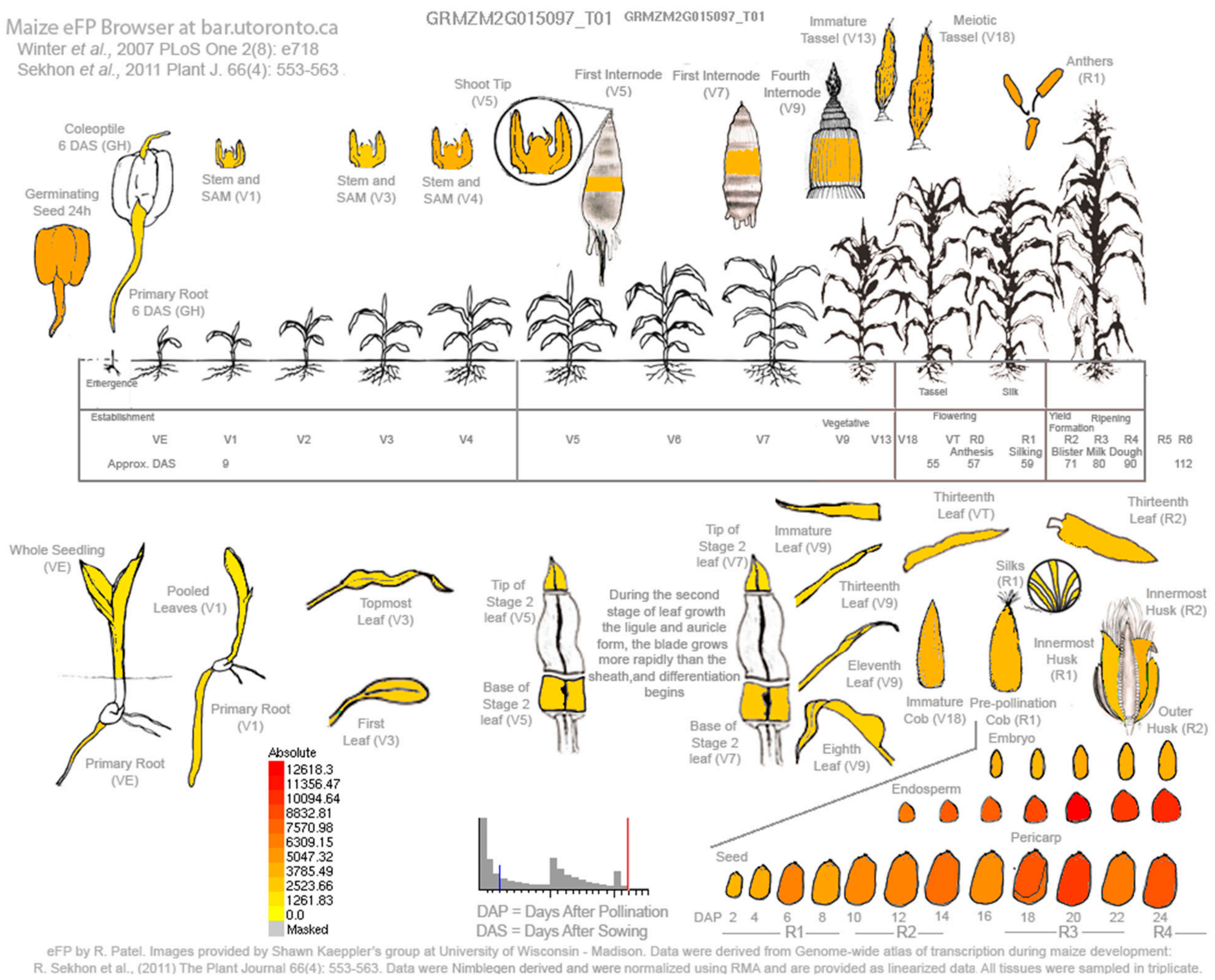

Supplemental Figure S6. Gene expression atlas showing tissue specific expression of CBBP/CPP2 (GRMZM2G015097) mainly in seed and low/no expression in vegetative tissues.

Supplemental Table S1. Expanded protein-protein interactions involving DDT4 primary PPIs. Uppercase gene names are inferred from *Arabidopsis thaliana* best BLAST hits. Sentence case gene names correspond to annotated maize gene models.

Gene	Maize Gene ID	Descriptive Name
	GRMZM2G007659	Cell division control protein
MMS21	GRMZM2G022065	E3 SUMO-protein ligase
	GRMZM2G025340	Structural maintenance of chromosomes protein 6B
	GRMZM2G027867	Condensin complex subunit 2
Spt6	GRMZM2G041697	RNA-Pol II transcription elongation factor
CHR10	GRMZM2G049168	Putative helicase
	GRMZM2G051894	ARM repeat superfamily protein
Afd1	GRMZM2G059037	Absence of first division1
Chr101a	GRMZM2G071025	Chromatin complex subunit A 106
	GRMZM2G074818	Sister chromatid cohesion 1 protein 3
	GRMZM2G093119	Sister chromatid cohesion 1 protein 4
	GRMZM2G096184	ARM repeat superfamily protein
	GRMZM2G099074	Embryo defective 1379
BUB1	GRMZM2G105750	Mitotic checkpoint serine/threonine-protein kinase
Ddt4	GRMZM2G128176	DNA binding
Mcm7	GRMZM2G139894	MCM family DNA helicase
MAGE	GRMZM2G142119	melanoma-associated antigen domain containing protein
ASHH1	GRMZM2G147619	Histone-lysine N-methyltransferase
	GRMZM2G149802	DNA polymerase (beta)
Cha101	GRMZM2G149802	Chromatin complex subunit A101
	GRMZM2G300624	Separase
	GRMZM2G318010	Aminopeptidase
	GRMZM2G326328	Condensin complex subunit 1
	GRMZM2G352431	Histone methyltransferase(H3-K4 specific)
	GRMZM2G363408	Cell division control protein; Belongs to the CDC6/cdc18 family
	GRMZM2G383623	Structural maintenance of chromosomes protein
	GRMZM2G421829	Sister chromatid cohesion 1 protein 4
	GRMZM2G440916	Structural maintenance of chromosomes protein 5
	GRMZM2G456570	Structural maintenance of chromosomes protein
	GRMZM2G470365	Sister chromatid cohesion 1 protein 2
MOM1	GRMZM2G472428	Helicase protein
Gtc102	GRMZM5G806358	FACT complex subunit SPT16
	GRMZM5G899800	Structural maintenance of chromosomes (SMC) family protein

Supplemental Table S2. 144 Proteins uniquely associated with *UAS::b1TR*.

Accession	Protein Name	Gene ID	Length (aa)
B4FBK3	Eukaryotic initiation factor 4A (Eukaryotic initiation factor 4A-2)	Zm00001d015251	410
K7UCZ5	Heat shock 70 kDa protein 6 chloroplastic	Zm00001d023802	703
Q84TL6	Legumin-like protein (RmlC-like cupins superfamily protein)	Zm00001d035597	363
B4FRM7	60S ribosomal protein L12 (60S ribosomal protein L12-3)	Zm00001d002450	166
A0A1D6MCX0	60S ribosomal protein L18a-2	Zm00001d039020	466
B4FB66	40S ribosomal protein S5-2	Zm00001d037929	199
A0A1D6QHF8	2-isopropylmalate synthase B	Zm00001d052472	661
C0P2Y6	Spermidine synthase 2	Zm00001d005159	318
B4FR90	Hyaluronan / mRNA binding family	Zm00001d043632	378
A0A1D6I0G0	Polyadenylate-binding protein (PABP)	Zm00001d019824	631
B6SGY5	RNA binding protein	Zm00001d012824	179
B6SZP7	Phosphoribulokinase	Zm00001d002454	381
A0A1D6LE84	EMBRYO DEFECTIVE 140	Zm00001d035065	539
B6TZV5	Uncharacterized protein	Zm00001d024471	71
C0P3M6	Basic endochitinase B	Zm00001d027525	259
A0A1D6GZI1	Uncharacterized protein	Zm00001d015138	4197
K7V6Z1	Putative patellin family protein	Zm00001d012254	556
A0A096S3Y8	DNA binding (DDT4)	Zm00001d022417	1712
A0A1D6JX93	Peroxisomal nicotinamide adenine dinucleotide carrier	Zm00001d028542	314
A0A1D6JG49	6,7-dimethyl-8-ribityllumazine synthase (DMRL synthase) (EC 2.5.1.78)	Zm00001d026476	240
B4FFJ0	Asparagine synthetase [glutamine-hydrolyzing] (EC 6.3.5.4)	Zm00001d045675	591
K7TNW2	Leucoanthocyanidin reductase	Zm00001d026370	343
C0PK04	Soluble inorganic pyrophosphatase 2	Zm00001d017770	201
B4F871	Protein DJ-1 homolog D (YLS5)	Zm00001d001881	387
A0A1D6FQW2	EMC1_C domain-containing protein	Zm00001d010413	866
A0A1D6GGS0	Ankyrin repeat family protein	Zm00001d013242	231
A0A1D6FLF3	Plasminogen activator inhibitor 1 RNA-binding protein	Zm00001d009740	376
B6TNK5	Uncharacterized protein	Zm00001d032250	226
A0A1D6MHU1	Clavaminate synthase-like protein	Zm00001d039517	355
B4FY74	Alpha/beta-Hydrolases superfamily protein	Zm00001d050198	264
A0A1D6EGC6	60 kDa jasmonate-induced protein	Zm00001d004568	198
B6UCW8	Ferredoxin-thioredoxin reductase, variable chain	Zm00001d017364	157
A0A1D6LUG1	Adrenodoxin-like protein 2 mitochondrial	Zm00001d037104	130
B4FUA7	Aldehyde dehydrogenase		478
K7U4C2	Uncharacterized protein	Zm00001d051823	333
B6TE60	Putative proteasome inhibitor	Zm00001d021719	301

A0A1D6JW03	ATP-dependent Clp protease ATP-binding subunit CLPT1 chloroplastic	Zm00001d028414	245

Supplemental Table 2 Continued.

Accession	Protein Name	Gene ID	Length (aa)
A0A1D6N0J1	Importin subunit alpha-6	Zm00001d042015	166
B6TBD4	Nascent polypeptide-associated complex subunit beta	Zm00001d048526	163
A0A1D6F9W9	Putative carboxylesterase 15	Zm00001d007908	342
A0A1D6G8G7	Mannose-1-phosphate guanylyltransferase 1	Zm00001d012395	302
B4FL28	Isovaleryl-CoA dehydrogenase mitochondrial	Zm00001d035475	390
A0A1D6MDV5	Carboxypeptidase (EC 3.4.16.-)	Zm00001d039146	453
A0A1D6J4J1	Germin-like protein	Zm00001d025059	227
A0A1D6MTX1	5-methylthioadenosine/S-adenosylhomocysteine deaminase	Zm00001d041069	137
A0A1D6GAX8	Chorismate mutase1	Zm00001d012674	365
A0A1D6LHI9	Beta-hexosaminidase (EC 3.2.1.52)	Zm00001d035598	588
O82725	DNA-directed RNA polymerase (EC 2.7.7.6)	Zm00001d046835	952
A0A1D6H396	Chloride channel protein	Zm00001d015702	648
B4FL26	TLD family protein (TLD-domain containing nucleolar protein)	Zm00001d001947	544
A0A1D6DVD2	Putative UDP-arabinopyranose mutase 5	Zm00001d001979	423
A0A1D6K0P5	FAD/NAD(P)-binding oxidoreductase family protein	Zm00001d028907	578
A0A1D6I2D1	Cystathionine gamma-synthase 1 chloroplastic	Zm00001d020141	263
A0A1D6ESR3	Uridine kinase (EC 2.7.1.48)	Zm00001d006084	404
A0A1D6K602	Putative ubiquitin conjugation factor E4	Zm00001d029561	877
K7TR40	Oxidoreductase family protein	Zm00001d024640	378
Q6R9K4	Uncharacterized protein orf105-a		105
A0A1D6MHD4	Agamous-like MADS-box protein AGL5	Zm00001d039434	262
K7TZE4	Uncharacterized protein	Zm00001d024186	196
C0PFX5	TIM-barrel signal transduction protein isoform 2	Zm00001d014524	737
A0A1D6P1B1	Beta-glucosidase 11	Zm00001d046210	424
A0A1D6F5I7	Subtilisin-like serine endopeptidase family protein	Zm00001d007319	659
A0A1D6MFW1	Phosphoacetylglucosamine mutase (PAGM) (EC 5.4.2.3) (Acetylglucosamine phosphomutase) (N-acetylglucosamine-phosphate mutase)	Zm00001d039361	624
A0A1D6N4R2	Uncharacterized protein	Zm00001d042515	487
B4G087	Adenine nucleotide alpha hydrolase-like superfamily protein	Zm00001d038351	164
B6TPA4	Carnitine racemase/ catalytic	Zm00001d038755	242
B4FKE8	Pi starvation-induced protein (Proteinase inhibitor propeptide)	Zm00001d038327	129
A0A1D6ES37	IRK-interacting protein	Zm00001d006025	607
B4FWN1	NADPH:quinone oxidoreductase	Zm00001d011845	210

A0A096SZL3	DNAJ heat shock N-terminal domain-containing protein	Zm00001d038879	338
A0A1D6N6I0	UDP-glycosyltransferase 91A1	Zm00001d042740	956
C4J9R0	PLAT domain-containing protein 3	Zm00001d003457	204

Supplemental Table 2 Continued.

Accession	Protein Name	Gene ID	Length (aa)
K7TVP5	Sugar isomerase (SIS) family protein	Zm00001d026095	215
A0A1D6FCA6	Putative uridine nucleosidase 2	Zm00001d008376	365
B4FQ24	Cytochrome P450 family 706 subfamily A polypeptide 5 (Flavonoid 3-monooxygenase)	Zm00001d032035	518
B6SQD4	Catalytic/ oxidoreductase, acting on NADH or NADPH	Zm00001d047888	91
K7W0U9	NB-ARC domain-containing protein	Zm00001d011737	519
Q42420	Protease inhibitor (Proteinase inhibitor) (Substilin /chymotrypsin-like inhibitor) (Subtilisin-chymotrypsin inhibitor CI-1B)	Zm00001d011080	73
B4FV70	Gibberellin 20-oxidase4	Zm00001d013725	349
A0A1D6FMB4	NifU-like protein 4 mitochondrial	Zm00001d009849	252
A0A1D6J864	Betaine aldehyde dehydrogenase 2 mitochondrial	Zm00001d025626	288
B7ZZ56	Glycosyltransferase (EC 2.4.1.-)	Zm00001d011649	480
A0A1D6QVF3	Uridine kinase	Zm00001d054105	527
K7TXI5	Chlorophyll a-b binding protein, chloroplastic	Zm00001d026599	248
A0A1D6JNX1	Cell division control protein 48 homolog D	Zm00001d027707	807
A0A1D6N6H1	Actin-7	Zm00001d042731	376
A0A1D6GPH0	Malate dehydrogenase (EC 1.1.1.37)	Zm00001d014030	312
A0A1D6MWD6	DEAD-box ATP-dependent RNA helicase 53	Zm00001d041480	599
B6TYX7	Polygalacturonase inhibitor 1	Zm00001d029583	284
B6T2N3	Peptide-methionine (R)-S-oxide reductase (EC 1.8.4.12)	Zm00001d037273	209
A0A1D6IYJ6	Putative leucine-rich repeat receptor-like protein kinase family protein	Zm00001d024291	581
A0A1D6LA43	UDP-glycosyltransferase 76C1	Zm00001d034692	486
A0A1D6HY75	Photosystem I reaction center subunit IV A	Zm00001d019518	136
A0A1D6NCP2	Golgin candidate 1	Zm00001d043502	704
A0A1D6JAW1	(+)-neomenthol dehydrogenase	Zm00001d025923	311
B6SZG7	Peptidylprolyl isomerase (EC 5.2.1.8)	Zm00001d046940	213
A0A1D6MR13	Type III polyketide synthase B	Zm00001d040479	398
A0A1D6LQX4	Uncharacterized protein	Zm00001d036756	99
Q9SLP6	Ferredoxin--NADP reductase, chloroplastic (FNR) (EC 1.18.1.2)	Zm00001d045575	355
B4FFA1	Oxidoreductase zinc-binding dehydrogenase family protein (Quinone oxidoreductase)	Zm00001d018418	329
A0A1D6EKW3	Uncharacterized protein	Zm00001d005170	357

A0A1D6NCB4	Trehalose-6-phosphate synthase6	Zm00001d043468	793
A0A1D6ITB4	Signal recognition particle 54 kDa protein chloroplastic	Zm00001d023431	564
A0A1D6JI62	Stem-specific protein TSJT1	Zm00001d026632	265
A0A1D6FA18	Alanine amino transferase8	Zm00001d007937	479
A0A1D6HPY9	ABC transporter F family member 3	Zm00001d018522	588
K7UVN6	Triose phosphate/phosphate translocator, chloroplastic	Zm00001d009028	404
B6U016	Senescence-associated protein DIN1	Zm00001d014816	191

Supplemental Table 2 Continued.

Accession	Protein Name	Gene ID	Length (aa)
A0A1D6GYN3	Protein LOW PSII ACCUMULATION 3 chloroplastic	Zm00001d015004	439
B6T4C2	CBS domain containing protein (OSIGBa0147B06.5 protein)	Zm00001d051908	222
A0A1D6G801	Uncharacterized protein	Zm00001d012287	206
A0A1D6KTS3	Nucleolin 2	Zm00001d032748	768
A0A1R3QF47	Chloroplast stem-loop binding protein of 41 kDa a chloroplastic	Zm00001d019177	449
A0A1D6MD76	Protease Do-like 1 chloroplastic	Zm00001d039059	481
A0A1D6N503	Trigger factor	Zm00001d042533	256
B4FJV4	Putative carboxylesterase 15	Zm00001d008634	331
A0A1D6NBM6	Callose synthase 11	Zm00001d043428	1745
A0A1D6LI73	D-glycerate 3-kinase chloroplastic	Zm00001d035737	436
B6TXY3	Quinone oxidoreductase-like protein (Quinone oxidoreductase-like protein At1g23740)	Zm00001d032332	386
A0A1D6MS70	Protein translocase subunit SECA1 chloroplastic	Zm00001d040686	1130
A0A1D6KFZ3	Cell-cell signaling protein csgA-like	Zm00001d031026	217
A0A1D6LKG8	Magnesium protoporphyrin IX methyltransferase chloroplastic	Zm00001d036046	324
A0A1D6N987	FAD-dependent oxidoreductase family protein	Zm00001d043147	369
A0A1D6N0U0	NADPH:quinone oxidoreductase	Zm00001d042061	202
B4FP01	Diphosphocytidyl methyl erythritol synthase1	Zm00001d042584	298
A0A1D6KLY8	Brachytic2	Zm00001d031871	1416
K7U772	Glutamyl-tRNA(Gln) amidotransferase subunit B, chloroplastic/mitochondrial (Glu-AdT subunit B) (EC 6.3.5.-)	Zm00001d052622	546
A0A1D6G430	Phosphatidylserine decarboxylase proenzyme 3	Zm00001d011827	595
B6TI70	Major myo-inositol transporter iolT (Putative polyol transporter 1)	Zm00001d023939	478
B4F887	S-adenosylmethionine carrier 1 chloroplastic/mitochondrial	Zm00001d017937	287
B4FMV4	Thioredoxin superfamily protein	Zm00001d005482	177
K7UIW6	Myosin heavy chain-related protein	Zm00001d025568	456

A0A1D6MXS5	Glutathione synthetase (GSH-S) (EC 6.3.2.3)	Zm00001d041710	475
B6TEU8	COP9 signalosome complex subunit 6	Zm00001d052848	333
A0A1D6FHV7	Uncharacterized protein	Zm00001d009141	173
A0A1D6L4R0	Protein TORNADO 1	Zm00001d034034	1048
A0A1D6HPC4	NADH dehydrogenase [ubiquinone] 1 alpha subcomplex subunit 9 mitochondrial	Zm00001d018479	342

Supplemental Table S3. Plasmid Constructs and Strains Used in the Yeast Transformation Experiment

Strain ID	Strain Type	Plasmid Description
KKY006	Positive control strain	*b1TR* bait + CBBP prey
KKY004	DDT4 Experimental strain	*b1TR* bait + DDT4 prey
KKY000	*b1TR*- negative control strain	Non-*b1TR* bait + DDT4 prey
YGY001	ZAG4 Experimental strain	*b1TR* bait + ZAG4 prey
YGY002	ZAG4ΔDNA binding domain	*b1TR* bait + ZAG4ΔBD prey
YGY003	*b1TR*- negative control strain	Non-*b1TR* bait + ZAG4 prey
KKY002	Negative control strain	Non-*b1TR* bait + CBBP prey

Supplemental Table S4. Transcription factor binding motif analysis of the b1TR.

Matrix ID	Family	Position	Strand	Similarity Score	Hit Sequence
TFmatrixID_0129	AT-Hook	125	-	1	AATTTttaa
TFmatrixID_0129	AT-Hook	140	-	1	AATTTttat
TFmatrixID_0129	AT-Hook	168	-	1	AATTTttaa
TFmatrixID_0129	AT-Hook	176	+	1	ataaAAATT
TFmatrixID_0129	AT-Hook	181	-	1	AATTTttac
TFmatrixID_0129	AT-Hook	214	+	1	taaaAAATT
TFmatrixID_0131	AT-Hook	9	+	1	cggtATAAA
TFmatrixID_0131	AT-Hook	209	+	1	taaaATAAA
TFmatrixID_0133	AT-Hook	183	-	1	TTTTTaccc
TFmatrixID_0133	AT-Hook	266	+	1	cggtAAAAA
TFmatrixID_0135	AT-Hook	141	-	1	ATTTTtat
TFmatrixID_0135	AT-Hook	176	+	1	ataAAAAT
TFmatrixID_0135	AT-Hook	192	-	1	ATTTTtat
TFmatrixID_0136	AT-Hook	210	+	1	aaaATAAA
TFmatrixID_0137	AT-Hook	385	+	1	aaaAATAT
TFmatrixID_0137	AT-Hook	636	+	1	aaaAATAT
TFmatrixID_0138	AT-Hook	210	+	1	aAAATAaa
TFmatrixID_0140	AT-Hook	383	+	1	aaaAAAAT
TFmatrixID_0140	AT-Hook	634	+	1	aaaAAAAT
TFmatrixID_0143	AT-Hook	171	+	1	ttttAATAAa
TFmatrixID_0143	AT-Hook	208	+	1	ttaaAATAAa
TFmatrixID_0143	AT-Hook	316	+	1	ctgcAATAAt
TFmatrixID_0143	AT-Hook	422	-	1	tTTATTaatt
TFmatrixID_0148	AT-Hook	215	+	1	aaAAAATt
TFmatrixID_0148	AT-Hook	384	+	1	aaAAAATa
TFmatrixID_0148	AT-Hook	635	+	1	aaAAAATa
TFmatrixID_0152	AT-Hook	125	-	1	AATTTtt
TFmatrixID_0152	AT-Hook	140	-	1	AATTTtt
TFmatrixID_0152	AT-Hook	168	-	1	AATTTtt
TFmatrixID_0152	AT-Hook	178	+	1	aaAAATT
TFmatrixID_0152	AT-Hook	181	-	1	AATTTtt
TFmatrixID_0152	AT-Hook	216	+	1	aaAAATT
TFmatrixID_0153	AT-Hook	141	-	1	ATTTTtat
TFmatrixID_0153	AT-Hook	176	+	1	ataAAAAT
TFmatrixID_0153	AT-Hook	192	-	1	ATTTTtat
TFmatrixID_0156	ARF;B3	752	-	1	gtTGTCGgg
TFmatrixID_0213	C2H2	30	-	1	cAGTGTt
TFmatrixID_0213	C2H2	276	+	1	aACACTg
TFmatrixID_0262	GATA	697	-	1	ggGATCAcg
TFmatrixID_0264	GATA	326	+	1	ccAGATCccc
TFmatrixID_0264	GATA	616	-	1	atgGATCTtg

TFmatrixID_0270	GATA	327	+	1	cAGATCccct
TFmatrixID_0278	TALE;Homeodomain	574	-	1	ggcTGTCAcg
TFmatrixID_0293	TALE	574	-	1	ggcTGTCAcg
TFmatrixID_0296	ZF-HD	338	+	1	tcTAATCcca
TFmatrixID_0301	TALE	575	-	1	gcTGTCAc
TFmatrixID_0311	Trihelix	182	+	1	attTTTACcc
TFmatrixID_0311	Trihelix	266	-	1	cgGTAAAaaa
TFmatrixID_0311	Trihelix	428	+	1	aatTTTACcc
TFmatrixID_0312	Trihelix	76	+	1	tttgGGTTAa
TFmatrixID_0317	Trihelix	76	+	1	tttgGGTTAa
TFmatrixID_0382	NAC	825	+	1	ggGTCAAgg
TFmatrixID_0417	GeBP	713	-	1	gtGGTCGgg
TFmatrixID_0419	TBP	458	+	1	aaTATAT
TFmatrixID_0445	WRKY	825	+	1	ggGTCAAg
TFmatrixID_0458	WRKY	824	+	1	cggGTCAAgg
TFmatrixID_0570	TBP	142	-	1	tTTTTAtaga
TFmatrixID_0580	AT-Hook	386	+	1	aAAATAt
TFmatrixID_0580	AT-Hook	637	+	1	aAAATAt
TF_motif_seq_0246	TALE	282	-	1	GGTCA
TF_motif_seq_0246	TALE	348	-	1	TGTCA
TF_motif_seq_0246	TALE	354	-	1	AGTCA
TF_motif_seq_0246	TALE	577	-	1	TGTCA
TF_motif_seq_0246	TALE	679	-	1	AGTCA
TF_motif_seq_0246	TALE	826	-	1	GGTCA
TF_motif_seq_0238	(Motif sequence only)	16	+	1	AAAGT
TF_motif_seq_0238	(Motif sequence only)	45	+	1	AAAGA
TF_motif_seq_0238	(Motif sequence only)	97	-	1	CCTTT
TF_motif_seq_0238	(Motif sequence only)	420	-	1	CCTTT
TF_motif_seq_0283	(Motif sequence only)	320	+	1	AATAAt
TF_motif_seq_0333	(Motif sequence only)	315	-	1	cCTGCA
TF_motif_seq_0333	(Motif sequence only)	782	-	1	cCTGCA
TF_motif_seq_0343	(Motif sequence only)	101	-	1	ttTGTTT
TFmatrixID_0146	AT-Hook	411	-	0.99	ATTATgtgt
TFmatrixID_0150	AT-Hook	386	+	0.99	aaAATATtaa
TFmatrixID_0150	AT-Hook	386	-	0.99	aaaATATTaa
TFmatrixID_0220	CAMTA	489	-	0.99	gatACGCGt
TFmatrixID_0220	CAMTA	492	+	0.99	aCGCGTagc
TFmatrixID_0221	CAMTA	489	+	0.99	gataCGCGT
TFmatrixID_0266	GATA	326	-	0.99	ccAGATCcc
TFmatrixID_0267	GATA	326	+	0.99	ccAGATCccc

TFmatrixID_0267	GATA	616	-	0.99	atgGATCTtg
TFmatrixID_0281	HD-ZIP	318	-	0.99	gcAATAAtc
TFmatrixID_0310	Trihelix	182	+	0.99	attTTTACcc
TFmatrixID_0310	Trihelix	266	-	0.99	cgGTAAAaaa
TFmatrixID_0310	Trihelix	428	+	0.99	aatTTTACcc
TFmatrixID_0316	Trihelix	76	+	0.99	tttgGGTTAa
TFmatrixID_0316	Trihelix	563	-	0.99	cTAACCaatg
TFmatrixID_0365	MYB-related	433	-	0.99	taCCCTAtcc
TFmatrixID_0372	MYB-related;Myb/SANT	435	+	0.99	cccTATCCa
TFmatrixID_0443	WRKY	825	+	0.99	ggGTCAAg
TFmatrixID_0466	WRKY	824	+	0.99	cggGTCAAgg
TFmatrixID_0569	TBP	11	+	0.99	gtATAAAagttgtgt
TFmatrixID_0093	Dof	41	+	0.98	agaTAAAGaat
TFmatrixID_0323	MYB-related	66	+	0.98	acaATATCca
TFmatrixID_0465	WRKY	825	+	0.98	ggGTCAAg
TFmatrixID_0548	Dof	41	+	0.98	agaTAAAGaat
TFmatrixID_0569	TBP	136	-	0.98	accaaattTTTATag
TFmatrixID_0585	TBP	10	+	0.98	ggtATAAAagt
TFmatrixID_0188	bZIP	572	-	0.97	gtggCTGTCa
TFmatrixID_0227	CPP	361	-	0.97	attTCAAAtc
TFmatrixID_0271	GATA	616	+	0.97	atgGATCTtg
TFmatrixID_0334	MYB-related	66	+	0.97	acaATATCc
TFmatrixID_0448	WRKY	824	+	0.97	cggGTCAAgga
TFmatrixID_0527	ERF;RAV	61	-	0.97	atgCAACAatat
TFmatrixID_0560	ERF;RAV	61	-	0.97	atgCAACAatat
TFmatrixID_0326	MYB-related	67	+	0.96	caaTATCCat
TFmatrixID_0449	WRKY	825	+	0.96	ggGTCAAg
TFmatrixID_0570	TBP	193	-	0.96	tTTTTAtggt
TFmatrixID_0585	TBP	141	-	0.96	attTTTATaga
TFmatrixID_0265	GATA	615	-	0.95	aatgGATCTt
TFmatrixID_0336	MYB-related;MYB	33	+	0.95	tgtTAGGTaga
TFmatrixID_0348	ARR-B	616	+	0.95	atggATCTTg
TFmatrixID_0570	TBP	10	+	0.95	ggtaTAAAAg
TF_motif_seq_0324	(Motif sequence only)	716	-	0.95	GTCGGg
TF_motif_seq_0324	(Motif sequence only)	755	-	0.95	GTCGGg
TFmatrixID_0130	AT-Hook	456	-	0.94	taaaTATATa
TFmatrixID_0144	AT-Hook	386	+	0.94	aaaaTATTAaat
TFmatrixID_0288	HD-ZIP	423	+	0.94	ttaTTAATttt
TFmatrixID_0154	AT-Hook	386	+	0.93	aAAATAtt
TFmatrixID_0287	HD-ZIP	423	+	0.93	ttaTTAATtt
TFmatrixID_0287	HD-ZIP	423	-	0.93	ttATTAAttt
TFmatrixID_0440	TCP	505	+	0.92	cGGACCat
TFmatrixID_0088	ERF;AP2	754	-	0.91	tGTCGGgc

TFmatrixID_0144	AT-Hook	420	+	0.91	ccttTATTAatt
TFmatrixID_0455	WRKY	824	+	0.91	cggGTCAAgga
TFmatrixID_0467	WRKY	824	+	0.91	cggGTCAAggatg
TFmatrixID_0491	TBP	451	+	0.91	tagtctaaaTATATagaacca
TFmatrixID_0572	TBP	451	+	0.91	tagtctaaaTATATagaacca
